# Personalized monitoring of circulating tumor DNA with a specific signature of trackable mutations after chimeric antigen receptor T-cell therapy in follicular lymphoma patients

**DOI:** 10.3389/fimmu.2023.1188818

**Published:** 2023-06-05

**Authors:** Ana Jiménez-Ubieto, Alejandro Martín-Muñoz, María Poza, Sara Dorado, Almudena García-Ortiz, Enrique Revilla, Pilar Sarandeses, Yanira Ruiz-Heredia, Tycho Baumann, Antonia Rodríguez, María Calbacho, Pilar Martínez Sánchez, José María Sánchez Pina, Alejandro Martín García-Sancho, Gloria Figaredo, Daniel Gil-Alós, Laura Rufián, Margarita Rodríguez, Laura Carneros, Carolina Martínez-Laperche, Mariana Bastos-Oreiro, Chongwu Wang, María-Teresa Cedena, Inmaculada Rapado, Paula de Toledo, Miguel Gallardo, Antonio Valeri, Rosa Ayala, Joaquín Martínez-López, Santiago Barrio

**Affiliations:** ^1^ Department of Hematology, Hospital Universitario 12 de Octubre, Instituto de Investigación Sanitaria Hospital 12 de Octubre (imas12), CNIO, CIBERONC, Madrid, Spain; ^2^ Altum sequencing Co., Madrid, Spain; ^3^ Computational Science Department, Carlos III University, Madrid, Spain; ^4^ Departamento de Anatomía Patológica, Hospital Universitario 12 de Octubre, Madrid, Spain; ^5^ Departamento de Medicina Nuclear, Hospital Universitario 12 de Octubre, Madrid, Spain; ^6^ Department of Hematology, Hospital Universitario de Salamanca, IBSAL, CIBERONC, Salamanca, Spain; ^7^ Hospital General Universitario Gregorio Marañón, Madrid, Spain; ^8^ Hosea Precision Medical Technology Co., Ltd., Weihai, Shangdong, China

**Keywords:** follicular lymphoma, ctDNA (circulating tumor DNA), NGS (Next-Generation Sequencing), minimal residual disease, monitoring, PET/CT ^18^F-FDG, CAR T-cell therapy

## Abstract

**Background:**

CART therapy has produced a paradigm shift in the treatment of relapsing FL patients. Strategies to optimize disease surveillance after these therapies are increasingly necessary. This study explores the potential value of ctDNA monitoring with an innovative signature of personalized trackable mutations.

**Method:**

Eleven FL patients treated with anti-CD19 CAR T-cell therapy were included. One did not respond and was excluded. Genomic profiling was performed before starting lymphodepleting chemotherapy to identify somatic mutations suitable for LiqBio-MRD monitoring. The dynamics of the baseline mutations (4.5 per patient) were further analyzed on 59 cfDNA follow-up samples. PET/CT examinations were performed on days +90, +180, +365, and every six months until disease progression or death.

**Results:**

After a median follow-up of 36 months, all patients achieved a CR as the best response. Two patients progressed. The most frequently mutated genes were CREBBP, KMT2D and EP300. Simultaneous analysis of ctDNA and PET/CT was available for 18 time-points. When PET/CT was positive, two out of four ctDNA samples were LiqBio-MRD negative. These two negative samples corresponded to women with a unique mesenteric mass in two evaluations and never relapsed. Meanwhile, 14 PET/CT negative images were mutation-free based on our LiqBio-MRD analysis (100%). None of the patients had a negative LiqBio-MRD test by day +7. Interestingly, all durably responding patients had undetectable ctDNA at or around three months after infusion. Two patients presented discordant results by PET/CT and ctDNA levels. No progression was confirmed in these cases. All the progressing patients were LiqBio-MRD positive before progression.

**Conclusion:**

This is a proof-of-principle for using ctDNA to monitor response to CAR T-cell therapy in FL. Our results confirm that a non-invasive liquid biopsy MRD analysis may correlate with response and could be used to monitor response. Harmonized definitions of ctDNA molecular response and pinpointing the optimal timing for assessing ctDNA responses are necessary for this setting. If using ctDNA analysis, we suggest restricting follow-up PET/CT in CR patients to a clinical suspicion of relapse, to avoid false-positive results.

## Introduction

Follicular lymphoma (FL) is the second most common non-Hodgkin lymphoma in developed countries (1). It is considered an indolent disorder with a relatively favorable course. When patients require treatment, several immuno-chemotherapeutic options are available (2–6). Modern treatments often achieve long remissions, with median survival rates approaching 20 years (7, 8). However, 15 - 20% of patients are primary refractory or progress during the first two years after first-line therapy (POD24). These patients have a poor outcome, with a 5-year overall survival (OS) between 38% and 50% (9, 10). A remitting, relapsing clinical course characterizes the disease, whose expected duration of response decreases with increasing previous therapeutic lines. Moreover, high-grade transformation (HT) into aggressive lymphoma occurs in around 3% of the patients yearly (11–13).

These multiple relapsing patients are a priority population with unmet needs for novel treatments. In this setting, several targeted therapies are rapidly being evaluated in patients with FL who need therapy in the third or further lines. These therapies include lenalidomide (14), inhibitors of phosphoinositide 3-kinase (PI3K) (15) or enhancer of zeste homolog 2 (EZH2) (16), autologous stem cell transplantation (17), anti-CD20/CD3 bispecific antibodies (18) and anti-CD19 chimeric antigen receptor (CAR) T-cell (CART) therapy (19, 20).. The highest rates of complete response (CR) and longest Progression-Free Survival (PFS) are achieved with anti-CD20/CD3 bispecific antibodies (18) and CART therapies (19, 20). Two CD19-directed CART therapies, which were evaluated in patients treated with at least two prior therapy lines, are approved for refractory/relapsing (R/R) FL, namely, axicabtageneciloleucel (axi-cel) and tisagenlecleucel (tisa-cel). The results of ZUMA-5 (19) and ELARA studies (20) led to the approval of axi-cel and tisa-cel, respectively, after demonstrating complete responses of 79% and 69% and median PFS at 18 months of 65.6% and 12 months of 85.5%, respectively. Nevertheless, we are not able yet to properly identify prospectively those patients who will benefit from this therapy.

Recently, it has been shown within FL patients treated with tisa-cel in the ELARA trial, that elevated tumor burden [TB; total metabolic tumor volume (TMTV) ≥240 cm^3^] at baseline (chemotherapy previous to lymphodepletion), POD24 after initial immuno-chemotherapy, and >4 nodal areas at inclusion were clinical factors that correlated significantly with lower efficacy (21). In the immuno-chemotherapy setting, fluorodeoxyglucose (FDG) positron emission computed tomography (PET/CT) imaging using the Deauville 5-point score (D5PS) is the gold-standard assessment for end-of-treatment response in FL. However, there are no standardized guidelines for imaging-based lymphoma patients’ follow-up after CART therapy. Nevertheless, in diffuse large B-cell lymphoma (DLBCL), some studies confirm that an early response 30 days after infusion seems to be predictive for both PFS and OS (22, 23). Nevertheless, current studies confirming this data are missing in FL. Recently, our group demonstrated that circulating tumor DNA (ctDNA) is an emerging biomarker able to risk stratify and assess treatment response in FL patients treated with first-line immuno-chemotherapy (24). In DLBCL, Quin Deng et al. investigated the role of early molecular response determined by plasma-derived cell-free DNA (cfDNA) in 22 patients treated with axi-cel and found that a variant allele frequency (VAF) declination within the first week of infusion was associated with ongoing CR by PET/CT at three months (25). Also, a Standford group, when evaluating ctDNA at day +28 after axi-cel, confirmed that a positive test was highly predictive for PFS and OS with a median PFS of 3.03 months versus not reached (NR) (P < 0.0001) and a median OS of 19.0 months versus NR (P < 0.008) (26). To our knowledge, no studies have used liquid biopsy NGS methods in FL patients treated with anti-CD19 CART therapies.

Therefore, in this study, we aim to analyze the response to therapy in FL patients using ultra-deep sequencing of ctDNA (LiqBio-MRD) combined with D5PS PET/CT, to identify early on patients who could benefit from CART therapy. Additionally, we aim to assess CART expansion correlating the CAR T-cells levels measured by Real-Time Quantitative PCR (qPCR) and Flow Cytometry (FCM).

## Materials/subjects and methods

### Patient cohort and study design

This study was designed as a prospective observational study. The cohort included 11 R/R FL patients treated with axi-cel (n=2) or tisa-cel (n=9) between July 2019 and August 2021 at the Hospital 12 de Octubre (H12O) in Madrid. All patients’ informed consent was obtained according to the Declaration of Helsinki. The study inclusion criteria were age >18 years, histological confirmation, PET/CT evaluation availability before and after the CART infusion, archival formalin-fixed paraffin-embedded (FFPE) obtained before CART infusion, and enough biological material in sequential samples. Patients unable to reach day +100 because of death, progression, or loss of follow-up were excluded from the analysis. A PFS event was defined as disease relapse or death. A progressor was defined as a participant who experienced a PFS event. A durable responder was defined as a participant who did not experience a PFS event with at least a 6-month follow-up. Treatment was started according to the Groupe d’Etude des Lymphomes Folliculaires criteria (27), and imaging examinations were performed on days +90, +180, +365, and every six months after that.

All patients’ DNA from lymph node biopsies and ctDNA were obtained before the CART infusion. Somatic mutations of these samples were selected as disease biomarkers for liquid biopsy minimal residual disease (LiqBio-MRD) analysis at follow-up time points. The following biological materials were analyzed: DNA from FFPE lymph node biopsies (n=10), ctDNA (n=6) at the time of starting lymphodepletion (pre-LD), and follow-up ctDNA samples after CART infusion (n=48). Responses were assessed by applying the Lugano criteria (28).

### DNA extraction

FFPE lymph node genomic DNA (gDNA) was extracted and quantified as previously described (24). For circulating-free DNA (cfDNA) extraction, 10 to 20 mL of peripheral blood (PB) was collected in EDTA tubes and processed in less than four hours at the H12O. The plasma was separated with two centrifugation steps at 1600 × g and 4500 × g, and stored at −80°C until further use. Purification, quantification and quality control (fragment size and gDNA contamination assessment) of cfDNA were performed as previously described (24).

### Baseline genotyping and LiqBio-MRD biomarker selection

Lymph node gDNA and plasma cfDNA baseline samples were screened for mutations with a short-length Ampliseq Custom Panel (Thermo Fisher Scientific). The panel, established as a routinary diagnosis tool at the H12O, was designed to cover all coding regions of 56 lymphoma-specific genes in FFPE samples (**Supplementary Table S1**). Samples were sequenced with an average coverage of 2,150x on an Ion S5 System platform (Life Technologies, Thermo Fisher Scientific). Then, the bioinformatic pipeline previously described (24) was applied for identifying and annotating variants, excluding potential deamination-related artifacts, and selecting the best biomarkers per patient (**Supplementary Table S2**).

### LiqBio-MRD methodology and bioinformatic pipeline

A median of 7.86 ng per mL of plasma (range 2 - 320 ng/mL) were obtained, being the mean amount of cfDNA from the initial 10 - 20 mL of PB of 104.94 ng (range 5.3 - 960 ng). All samples with a gDNA/cfDNA ratio greater than one, calculated using the bioanalyzer electropherogram, were excluded. An amplicon-multiplexed mini-panel was defined for every patient to detect the selected MRD biomarkers identified at diagnosis (**Supplementary Table S2**). The resulting libraries included three biological replicates per biomarker. They were sequenced on the Ion S5 System platform (Life Technologies, Thermo Fisher Scientific) with an estimated depth of 500,000× per amplicon, as previously described (24, 29, 30). The LiqBio-MRD test demonstrated a potential VAF sensibility below 10^−4^ (24). However, all potential MRD biomarkers were screened in triplicates of three gDNA samples obtained from healthy control donors. As a result, the limit of detection (LOD) of each marker was defined, allowing the exclusion of those with a LOD above 10^−4^ (24). The following bioinformatic pipeline for calculating the LiqBio-MRD value for each sample, including demultiplexing, false positive rate control, and biological and technical noise correction, was executed as previously described (24).

### PET/CT imaging

PET/CT scans were performed with a General Electric Discovery MI (GEDMI) Scanner or a Siemens Biograph 6 Scanner. PET/CT and CT images were acquired in the same session after injection of 2.5 - 3 MBq/kg ^18^F-FDG for the GEDMI Scanner and 4 - 5 MBq/kg ^18^F-FDG for the Siemens scanner. All follow-ups were performed in the GEDMI scanner. CT scans obtained with a low-dose protocol were used for attenuation correction of the PET/CT images. During follow-up, PET/CT scans were visually assessed according to the D5PS (^18^F-FDG uptake of any residual lesion), using mediastinal blood pool and liver uptake as reference settings. PET/CT was considered positive when D5PS was 4 or 5, and D5PS from 1 to 3 were classified as PET/CT negative (28). PET/CT was performed before the lymphodepletion treatment and during follow-up (days +90, +180, +365, and every 6 months until disease progression or death). Additionally, patients were closely monitored with a physical examination and routine laboratory tests. If new symptoms or laboratory changes were detected, an extra PET/CT was performed only.

### Detection of CAR T-cells through real-time qPCR

Absolute standard Curve Method (ACM) qPCR was performed on gDNA isolated from PB mononuclear cells (PBMCs). To determine the number of integrated anti-CD19 CART copies in CAR T-cells, 50 - 100 ng gDNA were amplified. The following primers sequences were used for the CART qPCR: Ψ forward (FP): 5’ CAGGACTCGGCTTGCTGAAG 3’ and Ψ reverse (RP): 5’ TCCCCCGCTTAATACTGACG 3’ and for the albumin: (FP): 5’ GCTGTCATCTCTTGTGGGCTG 3’ and (RP): 5’ ACTCATGGGAGCTGCTGGTTC 3’. For duplex qPCR assay, fluorogenic probes were 5′ labeled with 6-carboxyfluorescein (FAM) for Ψ sequence or VIC for albumin, and 3′ labeled with Black Hole Quencher-1 (BHQ-1). Thermal cycling for PCR experiments was performed in QuantStudio™ 5 Real-Time PCR System using the following amplification conditions: 2 minutes at 50°C, an initial heating of 20 seconds at 95°C, and 40 cycles of 1 second at 95°C and 20 seconds at 60°C. Primers were purchased from Sigma Aldrich, and probes and TaqMan Gene Expression Master Mix were purchased from Applied Biosystem.

### Detection of CAR T-cells through flow cytometry

Total CD3^+^ T-lymphocytes and anti-CD19 CAR T-cells population were detected in infused patients’ PB by incubating 200 - 300 µl of PB patients’ samples with 250 ng of FITC-rhCD19 (ACROBiosystems) and the antibody panel CD3-PECy7, CD4-APCCy7 and CD8-PerCP-Cy5.5 (Biolegend) at 4°C for 30 minutes in the dark. Erythrocytes lysis was then performed with ACK Lysing Buffer (Gibco). After two washing steps with PBS, samples were acquired using a FACS Canto II flow cytometer (BD Biosciences) with FACSDiva software (BD Biosciences), and data were analyzed using FlowJo v10 software (TreeStar).

### Statistical analysis

The Mann-Whitney U test was used to determine statistically significant differences in the obtained samples LiqBio-MRD values among the PET/CT-related categories. The Pearson correlation coefficient was used to determine the linear relationship between CAR T-cells levels quantified by FCM and qPCR. Both tests were performed using Python, with the Python package SciPy (version 1.10.1). P-values ≤0.05 were considered significant.

## Results

### Patients’ characteristics and predictive features before infusion

A total of 11 FL patients with samples available were treated with CART therapy in our institution. Nine received tisa-cel and two patients axi-cel. One patient refractory to tisa-cel was excluded from the analysis. Patient demographics and baseline characteristics of the remaining patients are summarized in **Table 1**. Briefly, the median age at the moment of infusion was 54 years (range 38 - 69), and six patients were male (60%). A median of three therapy lines were administrated before infusion (range 1 – 4). Nine patients were POD24 and nine patients (90%) were refractory to the previous immuno-chemotherapy regimen. Four patients (40%) had received an autologous stem cell transplantation.

**Table 1:** Baseline demographic and disease characteristics of all treated patients.

Parameter	Infused patients, n =10
**Median age (IQR), years**	54 (38-69)
**≥ 65 years, n**	1
**Male, n**	6
**Stage at study entry III-IV, n**	9
**Bone marrow involvement at study entry,n**	3
**Bulky disease at baseline, n**	2
**FLIPI high (≥3) at study entry, n**	5
**Median time since diagnosis of FL, months**	13-98 (43)
**Median no. of previous therapies (range)**	3 (1-4)
**POD24 from first anti-CD20 mAb-containing therapy, n**	9
**Refractory^a^ to first anti-CD20 mAb-containing therapy, n**	8
**Refractory^a^ disease to last line of therapy, n**	9
Previous antineoplastic therapy, n	
** Anti-CD20 mAb + alkylating agent (same or different regimen)**	10
** Antracycline**	10
** PI3K inhibitors**	3
** Lenalidomide**	1
**Previous autologous ASCT, n**	4

^a^Refractory is defined as failure to respond to previous treatment (SD/PD as best response) or PD within 6 months of previous therapy completion. mAb, monoclonal antibody; PS, performance score; ASCT, autologous stem cell transplantation.

Before infusion, three patients (27%) received optional antineoplastic bridging chemotherapy for stabilization based on immuno-chemotherapy. All patients received fludarabine and cyclophosphamide as lymphodepletion therapy. After a median follow-up of 36 months (range 11 - 50 months), all patients achieved a CR as the best response (10 patients achieved CR in the three months evaluation, and one patient achieved a partial (PR) at this point but converted to CR in the six months evaluation PET/CT). Two patients progressed after infusion (six and 12 months after infusion, respectively). Four patients presented cytokine release syndrome (grade 1-3), and one patient developed neurotoxicity (grade 2). Two patients died (one because of lymphoma progression and the other one due to breast cancer without evidence of lymphoma). None of the analytical or clinical characteristics analyzed were statistically associated with prognosis.

### Genotyping on lymph node and cfDNA before CART infusion

**Figure 1:**
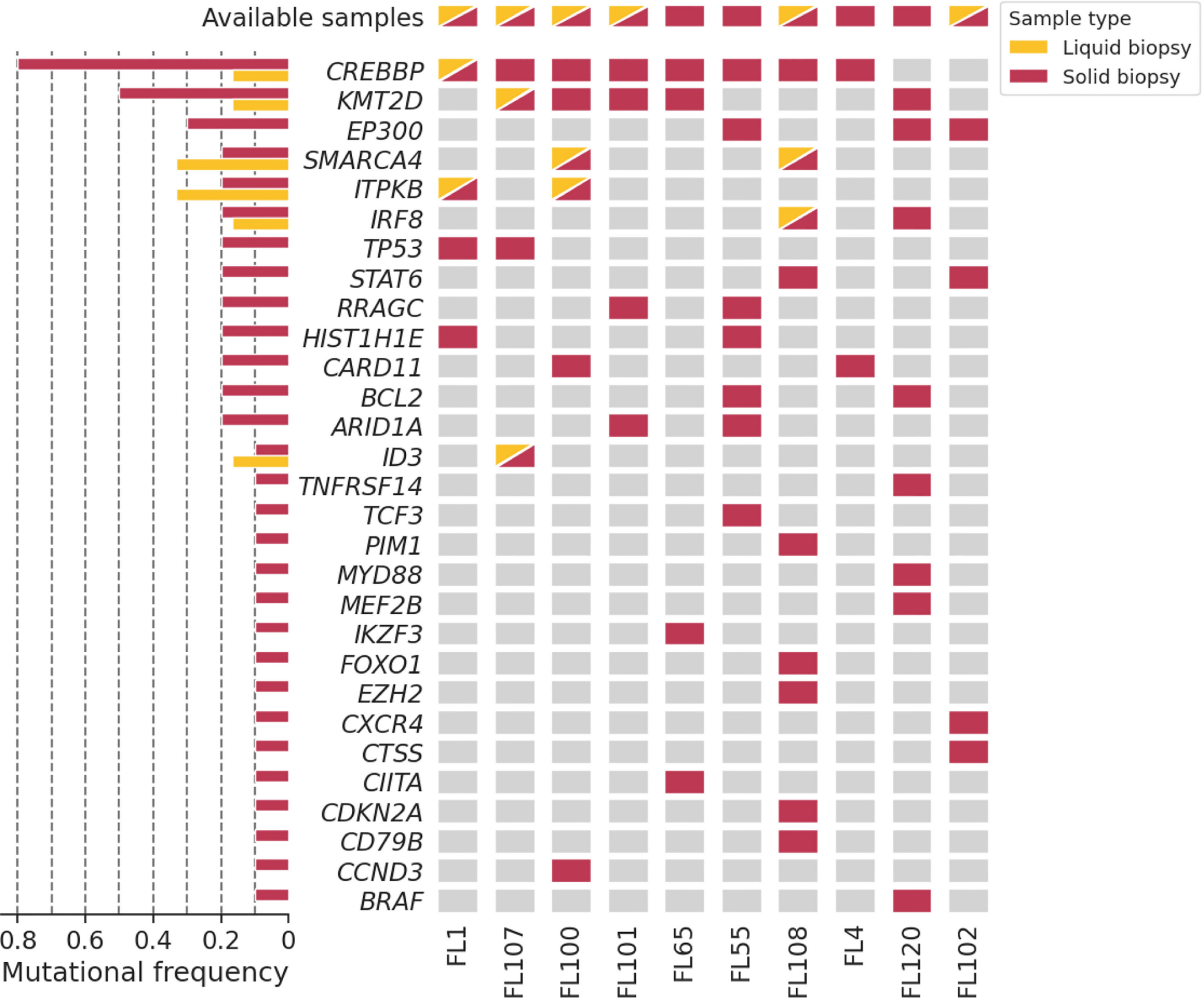
Oncoplot of the baseline genotyping of ten lymph node solid biopsy samples (red) and six plasma liquid biopsy samples (yellow). Patients are represented in the X-axis, and genes in the Y-axis.

Baseline genotyping with the targeted NGS panel was performed in ten gDNA lymph node samples and six cfDNA plasma samples. In the lymph node samples, 60 mutations were detected, with an average of six mutations per patient (range 3 - 10) and a mean VAF of 0.36 (range 0.0498 - 0.86). Eight mutations were detected in the cfDNA plasma samples, with an average of 1.334 mutations per patient (range 0 - 2) and a mean VAF of 0.423 (range 0.0306 - 0.5069). We found trackable mutations suitable for LiqBio-MRD monitoring in all patients. When only baseline cfDNA was considered, mutations were found in four out of six patients (66.6%). The most frequently mutated genes were CREBBP, KMT2D, and EP300 (**Figure 1**). In the 6 cases with available paired lymph node and plasma samples, eight mutations were identified in both fractions, 27 mutations were only detectable in the lymph node, and no mutations were only detected in plasma. No differences between the number of mutations or genes affected between long-term CR and R/R patients were observed.

### Circulating tumor DNA surveillance after CART therapy

**Figure 2:**
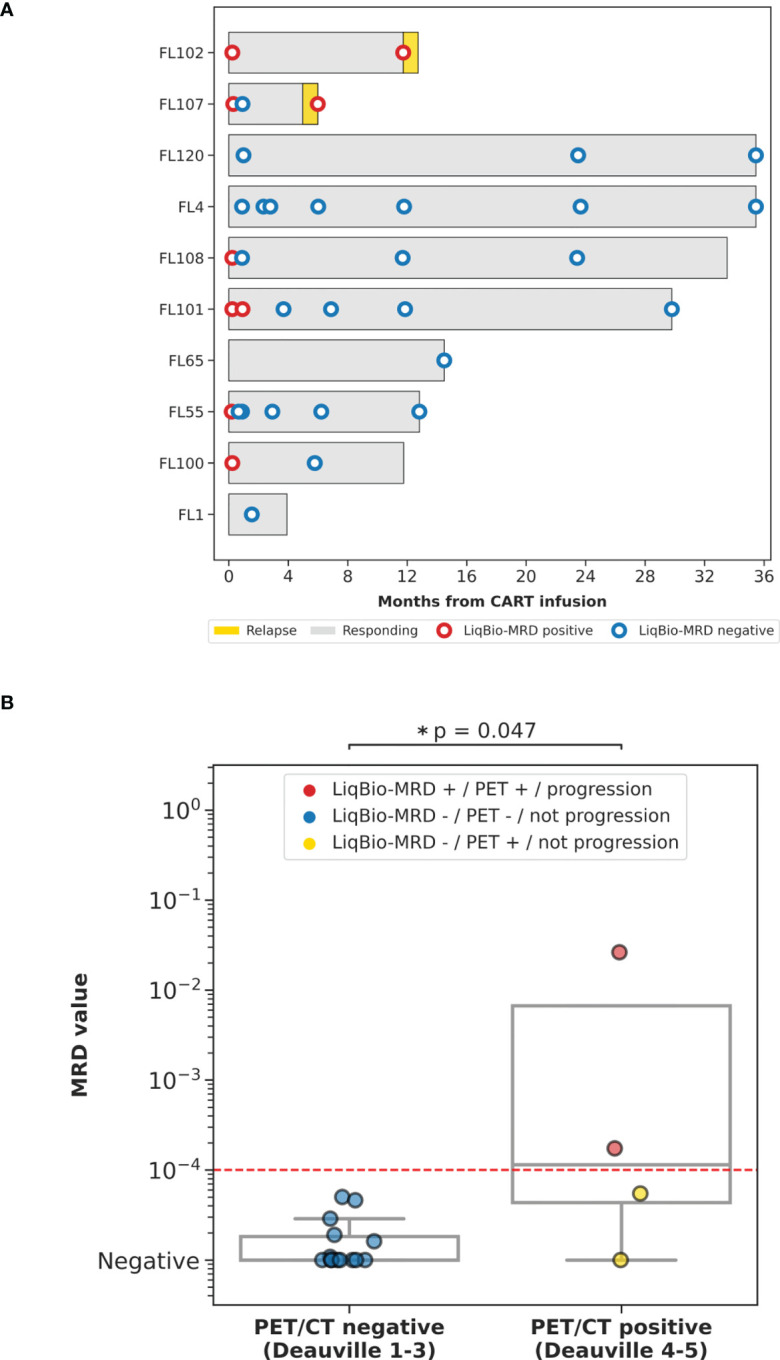
**(A)** Swimmer plot of the different follow-up time-points screened for all patients under CART treatment. **(B)** Correlation of Liqbio-MRD and PET/CT. The CR group included follow-ups with a D5PS of 1, 2, or 3 in nodal or extranodal sites with or without a residual mass. The non-CR group included PR cases with D5PS of 4 with reduced uptake compared with baseline and residual mass(es), and PD cases with a D5PS of 5 in any lesion with increased intensity of the ^18^F-FDG uptake from baseline * indicates p-value ≤ 0.05.

The dynamics of the baseline mutations were further analyzed on 59 cfDNA follow-up samples. The ctDNA was monitored until progression, death, or three years after therapy. Eleven samples were drawn before CART therapy infusion. One sample was not evaluable due to unsatisfactory sequencing performance, and 13 due to a low quantity of cfDNA (less than 30 ng). Finally, the distribution of the evaluable samples (n=45) was as follows: pre-infusion (n=10), day +7 (n=6), day +28 (n=8), month 3 (n=4), month 6 (n=5), year 1 (n=6), year 2 (n=3), and year 3 (n=3) (**Figure 2A**). On average, 4.5 mutations per patient (range 2 - 7) were selected as MRD biomarkers. The LiqBio-MRD dynamics for all patients are shown in **Supplementary Figure S1**. CtDNA was monitored for MRD in concordance with radiographic and clinical staging starting in month three. Simultaneous analysis of ctDNA and PET/CT was available for 18 time-points. To compare PET/CT and LiqBio-MRD results, we defined two groups based on the PET/CT data available when the liquid biopsy sample was collected. The first group (CR) (n=14) included follow-up samples with a D5PS of 1, 2, or 3 in nodal or extranodal sites with or without a residual mass. The non-CR group included PR (n=1) with a D5PS of 4 with reduced uptake compared with baseline and residual mass(es), and progressive disease (PD) (n=3) with a D5PS of 5 in any lesion with an increase in the intensity of FDG uptake from baseline. The LiqBio-MRD values were significantly lower for PET/CT-negative patients (**Figure 2B**). When PET/CT was positive (PR or PD), two out of four ctDNA samples were LiqBio-MRD negative (yellow points). These two negative samples corresponded to women with a unique mesenteric mass in two evaluations (PR month 3 and PD month 12). This patient (FL108) has not relapsed, with a median follow-up of 34 months. Meanwhile, 14 PET/CT negative samples appeared to be mutation-free based on our LiqBio-MRD analysis (100%).

**Figure 3:**
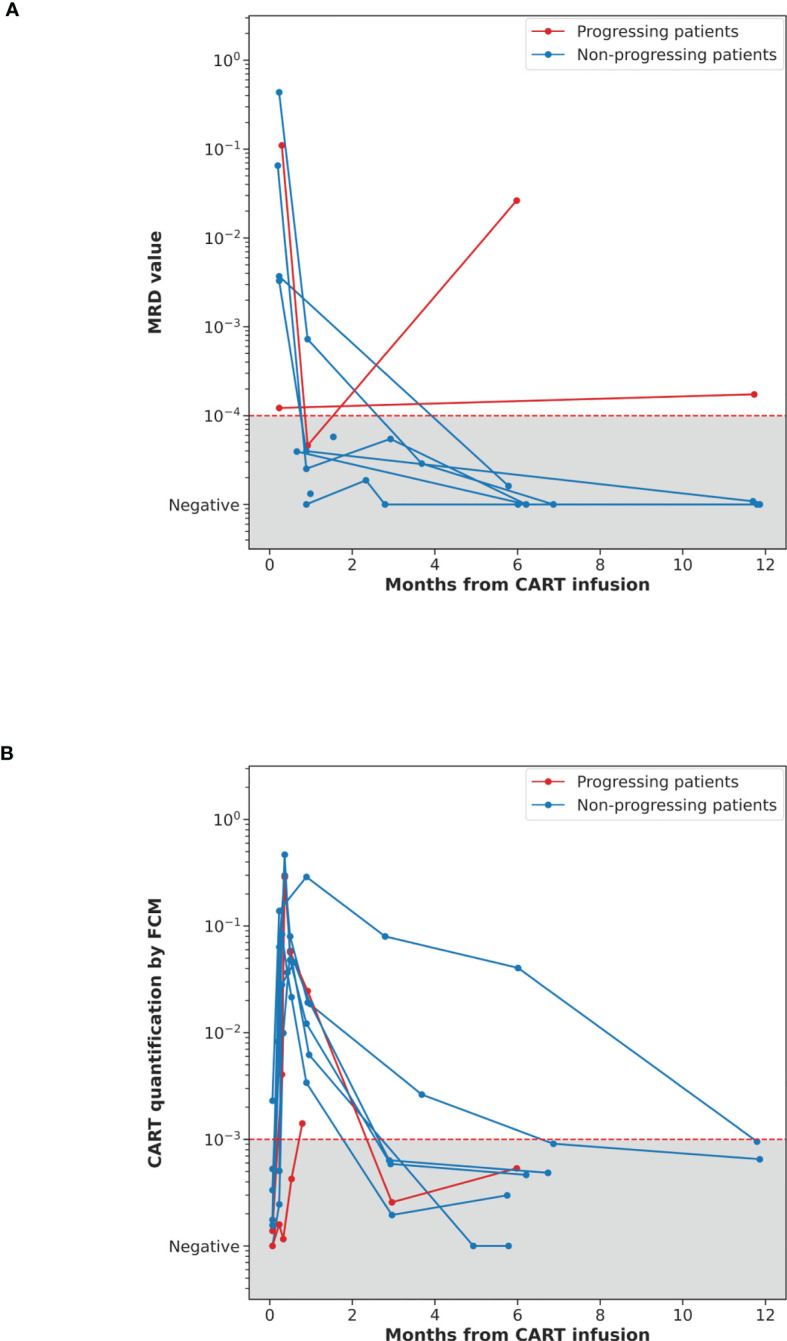
**(A)** Dynamics of cfDNA quantified by LiqBio-MRD in ten CART-treated FL patients. **(B)** Dynamics of CAR T-cell levels quantified by FCM in the same patients. The LiqBio-MRD values **(A)** and the CART FCM levels **(B)** for each follow-up data point (Y-axis) are plotted against the month since CAR T-cells infusion (X-axis). Patients that progressed are represented in red, CR and PR are represented in blue.

The ctDNA dynamics for the eight durable responders and two progressing patients are shown in **Figure 3A**. Before the first PET/CT evaluation (month three), 14 samples corresponding to nine patients were analyzed (day +7, n=6; and day +28, n=8). All day +7 samples (two corresponding to progression and four to long-term remission patients) were LiqBio-MRD positive. By day +28, in the group of patients without progression, seven ctDNA samples were available. Except for one sample, all the remaining were negative (**Figure 2A**). Interestingly, all durably responding patients had undetectable ctDNA at or around month three after CART infusion. Additionally, ctDNA was detected at or before radiographic relapse in the two progressing patients (**Figure 2A**).

The quantification of the CART levels by FCM is shown in **Figure 3B** and **Supplementary Figure S2**. CART expansion is observed in all patients, except for FL102, reaching maximum levels around day +7. In fact, FL102 is one of the progressing patients. Since day +28, levels decrease until becoming undetectable around month three, except for two patients whose CART levels have a slower decrease/reduction (undetectable at 7 and 12 months, respectively). FCM and qPCR CART quantification presented a strong correlation (**Supplementary Figure S3**).

### Dynamics of somatic mutations during the follow-up of FL patients

**Figure 4:**
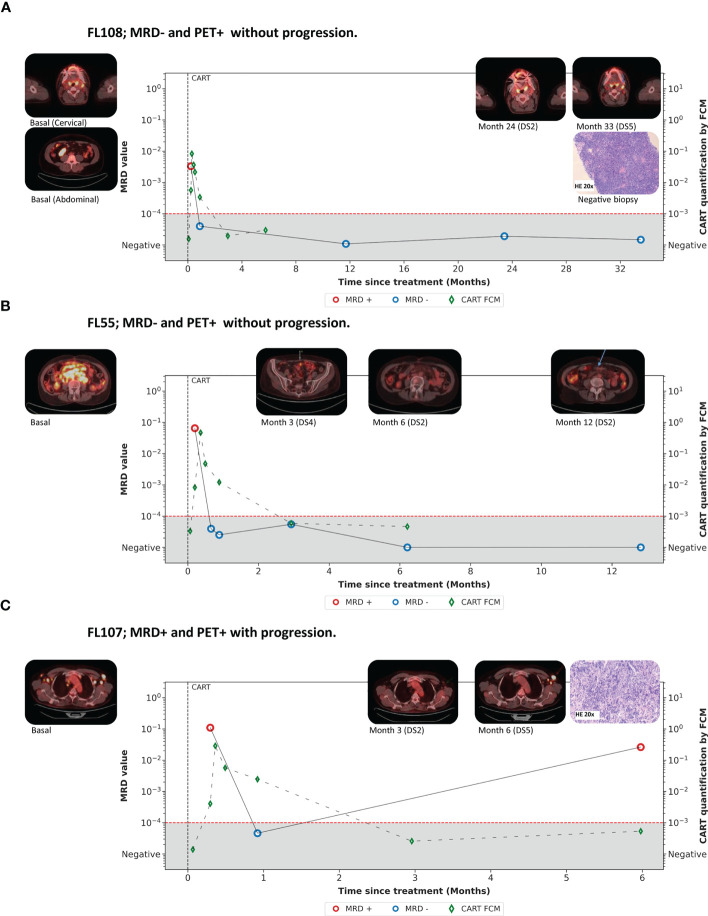
**(A-C)** Examples of the disease dynamics monitored by LiqBio-MRD.

Two patients presented discordant results by PET/CT and ctDNA levels. Patient FL108 showed ten mutations in the baseline lymph node and two in the baseline ctDNA (also detected in the lymph node). However, the seven mutations selected as MRD markers were only identified in the lymph node. Results from MRD assessment are available during the first two years after infusion (day +7, day +28, year 1, year 2). During this time, a response evaluation by PET/CT was performed on five time-points. During the first two years, all evaluations demonstrated a metabolic CR. However, a PET/CT performed 33 months after infusion suggested disease progression (D5PS of 5). There was a new onset of left cervical adenopathy in region IIA, whose metabolic activity was higher than liver parenchyma. Disease was not detected in any other side. The patient was asymptomatic. A biopsy was performed, excluding the lymphoma. Briefly, microscopic analysis showed a preserved lymph node architecture, the immunohistochemical stains found that B-cells were confined to germinal centers (CD10+, BCL-6+, BCL-2), and the PCR analysis of immunoglobulin heavy chain (IgH) was polyclonal. At this time-point, the LiqBio-MRD analysis persisted negative (**Figure 4A**).

Patient FL55 presented eight mutations in the lymph node (baseline cfDNA was unavailable). A total of four mutations (ARID1A, CREBP, RRACG, and BCL2) were selected as trackable during follow-up. LiqBio-MRD test was positive at day +7, but all the mutations were undetectable in the samples obtained by day +28, +36, month 3, month 6, and month 12 evaluations. She was diagnosed with a low-grade FL involving both sides of the diaphragm. The PET/CT evaluation performed at month three showed an excellent response to treatment but with a metabolic PR (D5PS of 4). This PR was explained by the persistence of a mesenteric uptake with a metabolic activity higher than the one of the liver parenchyma, complicating the interpretation of the imaging results. All the PET/CT subsequently performed confirmed the presence of a mesenteric striation with a metabolic activity lower than the liver parenchyma (D5PS of 2) (**Figure 4B**). This patient never progressed after 34 months of follow-up.

As indicated above, in this series, there were two progressing patients, of which only one patient (FL107) had a negative result by PET/CT and LiqBio-MRD and eventually progressed. Two somatic mutations in CREBBP and TP53 were found in this case. At day +7, the patient was LiqBio-MRD positive but became negative at day +28. PET/CT analysis performed three months after the CART infusion was confirmatory for CR. Nevertheless, a new PET/CT performed six months after CART infusion confirmed the progression of the disease in the left axillary region and the right costophrenic sinus (D5PS of 5) (**Figure 4C**). Interestingly, a LiqBio-MRD analysis performed during the PET/CT evaluation also detected the tracked mutations. In the second progressing patient (FL102), trackable mutations were CTSS, CXCR4, EP300, and STAT6. CtDNA analysis was available on day +7 and one year after infusion, and both LiqBio-MRD were positive. PET/CT analysis performed at day +100 and month six confirmed CR. Nevertheless, the one performed one year after infusion detected the progression of the disease. At this point, the LiqBio-MRD test could also detect the disease. It is also important to highlight that this is the only patient with poor expansion of the CAR T-cells population.

## Discussion

Anti-CD19 CART therapy is a new treatment modality for high-risk FL. However, major challenges include difficulties to prospectively identify later responders and to properly monitor disease progression after CART infusion. To our knowledge, this is the first study of serial ctDNA monitoring in FL patients with CD19-targeted CAR T-cell therapy. As previously described, our approach is based on the use of somatic mutations as disease biomarkers in a personalized way. First, we screened baseline lymph nodes and plasma samples to identify patient-specific biomarkers (24, 30) (**Figure 1**). Despite the limited number of patients, trackable somatic mutations suitable for MRD monitoring were found in all of them, confirming our previous results in FL patients treated with immuno-chemotherapy (24). There are a few studies in DLBCL (31, 32), classical Hodgkin lymphoma (33) and most recently in FL (24, 34) that confirm that a liquid biopsy MRD analysis by NGS based on somatic mutations is an emerging non-invasive test capable of detecting malignancy, monitoring treatment response and define treatment. However, there is not much data published about ctDNA in CAR T-cell therapy (35). In our FL cohort, two patients progressed by PET/CT after the infusion. Interestingly, we detected mutations at the time of progression in both (**Figures 2A**, **4**). Although it would be interesting to have serial ctDNA samples, our results confirm that disease progression in asymptomatic FL patients can be detected easily in a non-invasive way. In fact, in the eight non-relapsing patients, 25 LiqBio-MRD negative serial ctDNA samples confirm the absence of progression in the routine PET/CT performed. In DLBCL, large studies indicate that surveillance of ctDNA identifies patients at risk of recurrence before clinical evidence, both in the first line immuno-chemotherapy (32, 35) and, more recently, in patients receiving axi-cel, on whom ctDNA was detected at or before radiographic relapse in 29 of 30 (94%) relapsing patients (25).

Interestingly, all the patients of our cohort with available samples by day +7 (six of them durably responding) had detectable ctDNA. It is worth pointing out that CART expansion reached its peak precisely by day +7. This fact does not align with DLBCL undergoing CAR T-cell therapy, where ctDNA negativity by day +7 is a strong predictor of response and PFS (25, 26). This was already suggested in our previous results (24), indicating that, regardless of the treatment, FL presents a different dynamic than DLBCL (32), thus requiring the treatment more time to reach clearance of the tumor. As in our series, all durably responding patients had undetectable ctDNA at or around month three after CART infusion. Therefore, we hypothesize that serial ctDNA samples between day +7 and day +90 are critical in FL patients receiving CAR T-cell therapy to better understand the disease’s dynamics. This hypothesis should be tested in a prospective clinical trial.

Several groups have measured circulating CAR T-cell levels after infusion using qPCR or FCM. Some studies have reported that higher peak or cumulative CAR T-cell expansion is associated with durable disease control or severe neurotoxicity (36, 37). However, a similar relationship was not found in other studies (38). In our results, although maximum levels of expansion are reached around day +7, ctDNA persisted LiqBio-MRD positive in all the patients. To understand this issue, it should be studied in larger series of patients.

Two patients had PET/CT positive and LiqBio-MRD negative and no progression. In FL108 (**Figure 4A**), a PET/CT evaluation performed 33 months after infusion, a unique new onset of a left cervical adenopathy was found and therefore informed as disease progression. He had serial negative LiqBio-MRD tests. The exclusion of FL activity was done with a biopsy. Some reports confirm the lack of specificity of PET/CT in identifying lymphoma recurrence in the oropharynx and other cervical localization (39). Moreover, the progression in this patient happened during the COVID-19 pandemic. Interestingly, there are several cases of hypermetabolic adenopathy enlargement in patients with lymphoma related to the COVID-19 vaccine in a location where the disease was initially diagnosed (40). Although a biopsy was easily taken in this patient, LiqBio-MRD kinetics could help to define progression throughout different anatomical compartments when there is no chance of extracting a histological sample. In patient FL55 (**Figure 4B**), an excellent response to treatment in all the sites was obtained three months after infusion, but with the persistence of a mesenteric uptake with a metabolic activity higher than that of the liver parenchyma, complicating the interpretation of the imaging results. A biopsy was not accessible at this moment. The patient soon converted to CR. Interestingly, the ctDNA was LiqBio-MRD negative during the positive mesenteric uptake. Recently, Barrington et al. investigated the outcome of patients included in the GALLIUM study who did not achieve a metabolic CR (41). Only three out of 14 (21%) patients with mesenteric uptake as the only site of disease experienced progression. All together suggest that mesenteric ^18^F-FDG uptake is a common false positive finding at the end of induction in patients with FL who fail to achieve metabolic CR, most likely because of inflammatory uptake rather than residual lymphoma, especially in the context of CR at all other disease sites.

If confirmed in larger series, given that patients became LiqBio-MRD positive at or even before the time of disease relapse, serial ctDNA-based assessments may be a viable alternative to serial PET/CT imaging in asymptomatic patients. The use of PET/CT alone is hampered by its limited sensitivity and specificity, an unacceptable rate of false positives, and the interpretation of the results being highly dependent on the evaluating radiologist (41, 42). Specifically, in the context of CART or other immunotherapy treatments, imaging fails to capture pseudo-progression and to characterize the heterogeneous nature of the radiographically stable disease (43, 44). Interestingly, two of the patients in this series had false positive results of the PET/CT.

Our study has several limitations, including the small number of patients. Nevertheless, as the study was designed to provide an initial proof-of-principle for using ctDNA in monitoring response to CAR T-cell therapy in FL, we consider this to be a strength of the analysis. Although it seems mandatory to perform larger studies to confirm this preliminary data, our results demonstrate for the first time that NGS-based MRD quantification is feasible for monitoring treatment in liquid biopsies from FL patients undergoing CAR T-cell therapy.

As imaging-based response assessment typically occurs every two to three months, it is critical to develop disease biomarkers that are not only predictive of response, but can also give quick and early answers without invasive testing. This is of great interest considering the high rate of false positives PET/CT; even higher in the actual COVID-19 pandemic. In addition to the clinical value of longitudinal ctDNA assessment as a potential endpoint of CAR T-cell response, ctDNA molecular response can be used in early drug development. In this setting, ctDNA-based evaluation of the therapeutic effect of novel treatments would be faster and more accurately compared with radiographic imaging, ultimately expediting drug development.

In conclusion, non-invasive liquid biopsy MRD analysis may correlate with response and be used to monitor response in patients with relapsed FL treated with CAR T-cell therapy. Additionally, we suggest to investigate the value of ct DNA analysis in a larger number of FL patients undergoing CAR-T cell in order to investigate the value of PET/CT in CR patients. It is possible that follow-up PET/CT should only be required in CR patients with a clinical suspicion of relapse with the aim of avoiding false-positive results. Future studies may leverage this approach to identify patients requiring consolidative therapies following CART therapy. Nevertheless, harmonized definitions of ctDNA molecular response and pinpointing the optimal timing for assessing ctDNA responses are necessary to integrate liquid biopsies in precision immuno-oncology.

## Data availability statement

Publicly available datasets were analyzed in this study. This data can be found here: http://www.ncbi.nlm.nih.gov/bioproject/976692.

## Ethics statement

The studies involving human participants were reviewed and approved by Comite Etico Hospital 12 de Octubre. The patients/participants provided their written informed consent to participate in this study.

## Author contributions

AJ-U, AM-M, MP, and SB designed the research. LR, AG-O, ER, PS, and MR performed the experiments. AM-M, YR-H, SD, CW, and SB defined the bioinformatic pipeline and performed sequencing data analysis. AJ-U, MP, ER, TB, AR, MC, GF, M-TC, RA, and JM-L provided patient samples and clinical data. Author MG contributed to the conception, design and manuscript revisions. All authors analyzed and interpreted the data. AJ-U, AM-M, MP, and SB wrote the manuscript which was approved by all authors.

## References

[1] Anderson JR Armitage JO Weisenburger DD. Epidemiology of the non-hodgkin’s lymphomas: distributions of the major subtypes differ by geographic locations. Ann Oncol (1998) 9:717–20. doi: 10.1023/A:1008265532487 9739436

[2] Flinn IW van der Jagt R Kahl BS Wood P Hawkins TE Macdonald D. Randomized trial of bendamustine-rituximab or r-CHOP/R-CVP in first-line treatment of indolent NHL or MCL: the BRIGHT study key points. McKesson Specialty Health/US Oncol Res (2014) 9. doi: 10.1182/blood-2013-11-531327 PMC4260975 24591201

[3] Hiddemann W Kneba M Dreyling M Schmitz N Lengfelder E Schmits R. Frontline therapy with rituximab added to the combination of cyclophosphamide, doxorubicin, vincristine, and prednisone (CHOP) significantly improves the outcome for patients with advanced-stage follicular lymphoma compared with therapy with CHOP alone: results of a prospective randomized study of the German low-grade lymphoma study group. Blood (2005) 106. doi: 10.1182/blood-2005-01-0016 16123223

[4] Morschhauser F Fowler NH Feugier P Bouabdallah R Tilly H Palomba ML. Rituximab plus lenalidomide in advanced untreated follicular lymphoma. New Engl J Med (2018) 379(10):934–47. doi: 10.1056/NEJMoa1805104 PMC11003525 30184451

[5] Marcus R Davies A Ando K Klapper W Opat S Owen C. Obinutuzumab for the first-line treatment of follicular lymphoma. New Engl J Med (2017) 377(14):1331–44. doi: 10.1056/NEJMoa1614598 28976863

[6] Bachy E Seymour JF Feugier P Offner F López-Guillermo A Belada D. Sustained progression-free survival benefit of rituximab maintenance in patients with follicular lymphoma: long-term results of the PRIMA study. J Clin Oncol (2019) 37(31):2815–24. doi: 10.1200/JCO.19.01073 PMC6823890 31339826

[7] Cheah CY Chihara D Ahmed M Davis RE Nastoupil LJ Phansalkar K. Factors influencing outcome in advanced stage, low-grade follicular lymphoma treated at MD Anderson cancer center in the rituximab era. Ann Oncol (2016) 27(5):895–901. doi: 10.1093/annonc/mdw026 26802151

[8] Jiménez-Ubieto A Grande C Caballero D Yáñez L Novelli S Hernández-Garcia MT. Autologous stem cell transplantation for follicular lymphoma: favorable long-term survival irrespective of pretransplantation rituximab exposure. Biol Blood Marrow Transplantation. (2017) 23(10):1631–40. doi: 10.1016/j.bbmt.2017.05.021 28533060

[9] Jiménez-Ubieto A Grande C Caballero D Yáñez L Novelli S Hernández MT. Progression-free survival at 2 years post-autologous transplant: a surrogate end point for overall survival in follicular lymphoma. Cancer Med (2017) 6(12):2766–74. doi: 10.1002/cam4.1217 PMC5727300 29076254

[10] Casulo C Dixon JG Le-Rademacher J Hoster E Hochster HS Hiddemann W. Validation of POD24 as a robust early clinical end point of poor survival in FL from 5225 patients on 13 clinical trials. Blood (2022) 139(11):1684–93. doi: 10.1182/blood.2020010263 PMC9974165 34614146

[11] Pasqualucci L Khiabanian H Fangazio M Vasishtha M Messina M Holmes AB. Genetics of follicular lymphoma transformation. Cell Rep (2014) 6(1):130–40. doi: 10.1016/j.celrep.2013.12.027 PMC4100800 24388756

[12] Sarkozy C Trneny M Xerri L Wickham N Feugier P Leppa S. Risk factors and outcomes for patients with follicular lymphoma who had histologic transformation after response to first-line immunochemotherapy in the PRIMA trial. J Clin Oncol (2016) 34(22):2575–82. doi: 10.1200/JCO.2015.65.7163 27298402

[13] Sorigue M Mercadal S Alonso S Fernández-Álvarez R García O Moreno M. Refractoriness to immunochemotherapy in follicular lymphoma: predictive factors and outcome. Hematol Oncol (2017) 35(4):520–7. doi: 10.1002/hon.2378 28156010

[14] Leonard JP Trneny M Izutsu K Fowler NH Hong X Zhu J. AUGMENT: a phase III study of lenalidomide plus rituximab versus placebo plus rituximab in relapsed or refractory indolent lymphoma. J Clin Oncol (2019) 37(14):1188–99. doi: 10.1200/JCO.19.00010 PMC7035866 30897038

[15] Gopal AK Kahl BS de Vos S Wagner-Johnston ND Schuster SJ Jurczak WJ. PI3Kδ inhibition by idelalisib in patients with relapsed indolent lymphoma. New Engl J Med (2014) 370(11):1008–18. doi: 10.1056/nejmoa1314583 PMC4039496 24450858

[16] Morschhauser F Tilly H Chaidos A McKay P Phillips T Assouline S. Tazemetostat for patients with relapsed or refractory follicular lymphoma: an open-label, single-arm, multicentre, phase 2 trial. Lancet Oncol (2020) 21(11):1433–42. doi: 10.1016/S1470-2045(20)30441-1 PMC8427481 33035457

[17] Jiménez-Ubieto A Grande C Caballero D Yáñez L Novelli S Hernández-Garcia MT. Autologous stem cell transplantation may be curative for patients with follicular lymphoma with early therapy failure who reach complete response after rescue treatment. Hematol Oncol (2018) 36(5):765–72. doi: 10.1002/hon.2553 30129233

[18] Budde LE Sehn LH Matasar M Schuster SJ Assouline S Giri P. Safety and efficacy of mosunetuzumab, a bispecific antibody, in patients with relapsed or refractory follicular lymphoma: a single-arm, multicentre, phase 2 study. Lancet Oncol (2022) 23(8):1055–65. doi: 10.1016/S1470-2045(22)00335-7 35803286

[19] Jacobson CA Chavez JC Sehgal AR William BM Munoz J Salles G. Axicabtagene ciloleucel in relapsed or refractory indolent non-Hodgkin lymphoma (ZUMA-5): a single-arm, multicentre, phase 2 trial. Lancet Oncol (2022) 23(1):91–103. doi: 10.1016/S1470-2045(21)00591-X 34895487

[20] Schuster SJ Thieblemont C Hale Fowler N Dickinson M Dreyling M Martinez-Lopez J. Tisagenlecleucel in adult relapsed or refractory follicular lymphoma: the phase 2 ELARA trial. Nat Med (2021) 28:325–32. doi: 10.1038/s41591-021-01622-0 34921238

[21] Dreyling M Dickinson M Martínez-López J. (2022). Long-term clinical outcomes and correlative efficacy analyses in patients (Pts) with Relapsed/Refractory follicular lymphoma (r/r FL) treated with tisagenlecleucel in the elara trial, in: 64th ASH Annual Meeting and Exposition, . ASH.

[22] Georgi TW Kurch L Franke GN Jentzsch M Schwind S Perez-Fernandez C. Prognostic value of baseline and early response FDG-PET/CT in patients with refractory and relapsed aggressive b-cell lymphoma undergoing CAR-T cell therapy. J Cancer Res Clin Oncol (2023). doi: 10.1007/s00432-023-04587-4 PMC10356653 36662305

[23] Kuhnl A Roddie C Kirkwood AA Menne T Cuadrado M Marzolini MAV. Early FDG-PET response predicts CAR-T failure in large b-cell lymphoma. Blood Adv (2022) 6(1):321–6. doi: 10.1182/bloodadvances.2021005807 PMC8753214 34700342

[24] Jiménez-Ubieto A Poza M Martin-Muñoz A Ruiz-Heredia Y Dorado S Figaredo G. Real-life disease monitoring in follicular lymphoma patients using liquid biopsy ultra-deep sequencing and PET/CT. Leukemia (2023) 37(3):659–69. doi: 10.1038/s41375-022-01803-x 36596983

[25] Deng Q Han G Puebla-Osorio N Ma MCJ Strati P Chasen B. Characteristics of anti-CD19 CAR T cell infusion products associated with efficacy and toxicity in patients with large b cell lymphomas. Nat Med (2020) 26(12):1878–87. doi: 10.1038/s41591-020-1061-7 PMC8446909 33020644

[26] Frank MJ Hossain NM Bukhari A Dean E Spiegel JY Claire GK. Monitoring of circulating tumor DNA improves early relapse detection after axicabtagene ciloleucel infusion in Large b-cell lymphoma: results of a prospective multi-institutional trial. J Clin Oncol (2021) 39(27):3034–43. doi: 10.1200/JCO.21.00377 PMC10166351 34133196

[27] Brice P Bastion Y Lepage E Brousse N Haïoun C Moreau P. Comparison in low-tumor-burden follicular lymphomas between an initial no-treatment policy, prednimustine, or interferon alfa: a randomized study from the groupe d’Etude des lymphomes folliculaires. groupe d’Etude des lymphomes de l’Adulte. J Clin Oncol (1997) 15(3):1110–7. doi: 10.1200/JCO.1997.15.3.1110 9060552

[28] Cheson BD Fisher RI Barrington SF Cavalli F Schwartz LH Zucca E. Recommendations for initial evaluation, staging, and response assessment of Hodgkin and non-Hodgkin lymphoma: the lugano classification. J Clin Oncol (2014) 32(27):3059–67. doi: 10.1200/JCO.2013.54.8800 PMC4979083 25113753

[29] Sánchez R Dorado S Ruíz-Heredia Y Martín-Muñoz A Rosa-Rosa JM Ribera J. Detection of kinase domain mutations in BCR::ABL1 leukemia by ultra-deep sequencing of genomic DNA. Sci Rep (2022) 12(1):1–11. 35906470 10.1038/s41598-022-17271-3 PMC9338264

[30] Onecha E Linares M Rapado I Ruiz-Heredia Y Martinez-Sanchez P Cedena T. A novel deep targeted sequencing method for minimal residual disease monitoring in acute myeloid leukemia. Haematologica (2019) 104(2):288–96. doi: 10.3324/haematol.2018.194712 PMC6355493 30093399

[31] Kurtz DM Soo J Co Ting Keh L Alig S Chabon JJ Sworder BJ. Enhanced detection of minimal residual disease by targeted sequencing of phased variants in circulating tumor DNA. Nat Biotechnol (2021) 39(12):1537–47. doi: 10.1038/s41587-021-00981-w PMC8678141 34294911

[32] Kurtz DM Scherer F Jin MC Soo J Craig AFM Esfahani MS. Circulating tumor DNA measurements as early outcome predictors in diffuse Large b-cell lymphoma. J Clin Oncol (2018) 36(28):2845–53. doi: 10.1200/JCO.2018.78.5246 PMC6161832 30125215

[33] Spina V Bruscaggin A Cuccaro A Martini M Di Trani M Forestieri G. Circulating tumor DNA reveals genetics, clonal evolution, and residual disease in classical Hodgkin lymphoma. Blood (2018) 131(22):2413–25. doi: 10.1182/blood-2017-11-812073 29449275

[34] Fernández-Miranda I Pedrosa L Llanos M Franco FF Gómez S Martín-Acosta P. Monitoring of circulating tumor DNA predicts response to treatment and early progression in follicular lymphoma: results of a prospective pilot study. Clin Cancer Res (2023) 29(1):209–20. doi: 10.1158/1078-0432.CCR-22-1654 PMC9811164 36269794

[35] Roschewski M Dunleavy K Pittaluga S Moorhead M Pepin F Kong K. Circulating tumour DNA and CT monitoring in patients with untreated diffuse large b-cell lymphoma: a correlative biomarker study. Lancet Oncol (2015) 16(5):541–9. doi: 10.1016/S1470-2045(15)70106-3 PMC4460610 25842160

[36] Locke FL Ghobadi A Jacobson CA Miklos DB Lekakis LJ Oluwole OO. Long-term safety and activity of axicabtagene ciloleucel in refractory large b-cell lymphoma (ZUMA-1): a single-arm, multicentre, phase 1–2 trial. Lancet Oncol (2019) 20(1):31–42. doi: 10.1016/S1470-2045(18)30864-7 30518502 PMC6733402

[37] Locke FL Rossi JM Neelapu SS Jacobson CA Miklos DB Ghobadi A. Tumor burden, inflammation, and product attributes determine outcomes of axicabtagene ciloleucel in large b-cell lymphoma. Blood Adv (2020) 4(19):4898–911. doi: 10.1182/bloodadvances.2020002394 PMC7556133 33035333

[38] Neelapu SS Locke FL Bartlett NL Lekakis LJ Miklos DB Jacobson CA. Axicabtagene ciloleucel CAR T-cell therapy in refractory Large b-cell lymphoma. New Engl J Med (2017) 377(26):2531–44. doi: 10.1056/NEJMoa1707447 PMC5882485 29226797

[39] West JD Kim ME Lapalma DM Vergara-Lluri M Conti P Chambers TN. 18FDG-PET/CT specificity for the detection of lymphoma recurrence in the tonsils. OTO Open (2021) 5(4):2473974X211059081. doi: 10.1177/2473974X211059081 PMC8600558 34805720

[40] Landete E Gómez-Fernández I González-Gascón-y-Marín I Durán-Barquero C Churruca J Infante MS. Hypermetabolic abdominal and cervical lymph nodes mimicking Hodgkin lymphoma relapse on FDG PET/CT after adenovirus-vectored COVID-19 vaccine. Hum Vaccin Immunother (2021) 17(12):5129–32. doi: 10.1080/21645515.2021.2008215 PMC8903903 34920695

[41] Barrington SF Mir F El-Galaly TC Knapp A Nielsen TG Sahin D. Follicular lymphoma treated with first-line immunochemotherapy: a review of PET/CT in patients who did not achieve a complete metabolic response in the GALLIUM study. J Nucl Med (2022) 63(8):1149. doi: 10.2967/jnumed.121.262869 34857656 PMC9364340

[42] Ansell SM Armitage JO. Positron emission tomographic scans in lymphoma: convention and controversy. Mayo Clin Proc (2012) 87(6):571–80. doi: 10.1016/j.mayocp.2012.03.006 PMC3498383 22677077

[43] Sortais C Cordeil S Bourbon E Idlhaj M Ferrant E Safar V. Flare-up phenomenon or pseudoprogression after CAR T-cell infusion in non-Hodgkin aggressive lymphomas. Leuk Lymphoma (2022), 1–5. doi: 10.1080/10428194.2022.2161304 36573418

[44] Cheson BD Ansell S Schwartz L Gordon LI Advani R Jacene HA. Refinement of the lugano classification lymphoma response criteria in the era of immunomodulatory therapy. Blood (2016) 128(21):2489–96. doi: 10.1182/blood-2016-05-718528 27574190

