# Corrigendum: Personalized monitoring of circulating tumor DNA with a specific signature of trackable mutations after chimeric antigen receptor T-cell therapy in follicular lymphoma patients

**DOI:** 10.3389/fimmu.2023.1243241

**Published:** 2023-08-16

**Authors:** Ana Jiménez-Ubieto, Alejandro Martín-Muñoz, María Poza, Sara Dorado, Almudena García-Ortiz, Enrique Revilla, Pilar Sarandeses, Yanira Ruiz-Heredia, Tycho Baumann, Antonia Rodríguez, María Calbacho, Pilar Martínez Sánchez, José María Sánchez Pina, Alejandro Martín García-Sancho, Gloria Figaredo, Laura Rufián, Margarita Rodríguez, Laura Carneros, Carolina Martínez-Laperche, Mariana Bastos-Oreiro, Chongwu Wang, María-Teresa Cedena, Inmaculada Rapado, Paula de Toledo, Miguel Gallardo, Antonio Valeri, Rosa Ayala, Joaquín Martínez-López, Santiago Barrio

**Affiliations:** ^1^ Department of Hematology, Hospital Universitario 12 de Octubre, Instituto de Investigación Sanitaria Hospital 12 de Octubre (imas12), CNIO, CIBERONC, Madrid, Spain; ^2^ Altum sequencing Co., Madrid, Spain; ^3^ Computational Science Department, Carlos III University, Madrid, Spain; ^4^ Departamento de Anatomía Patológica, Hospital Universitario 12 de Octubre, Madrid, Spain; ^5^ Departamento de Medicina Nuclear, Hospital Universitario 12 de Octubre, Madrid, Spain; ^6^ Department of Hematology, Hospital Universitario de Salamanca, IBSAL, CIBERONC, Salamanca, Spain; ^7^ Hospital General Universitario Gregorio Marañón, Madrid, Spain; ^8^ Hosea Precision Medical Technology Co., Ltd., Weihai, Shangdong, China

**Keywords:** follicular lymphoma, ctDNA (circulating tumor DNA), NGS (Next-Generation Sequencing), minimal residual disease, monitoring, PET/CT 18F-FDG, CAR T-cell therapy

In the published article, there was an error in the author list, and author Miguel Gallardo was erroneously excluded. The corrected author list appears below:

Ana Jiménez-Ubieto^1*†^, Alejandro Martı´n-Muñoz^1,2†^,María Poza^1^, Sara Dorado^2,3^, Almudena García-Ortiz^1^, Enrique Revilla^4^, Pilar Sarandeses^5^, Yanira Ruiz-Heredia^1,2^, Tycho Baumann^1^, Antonia Rodríguez^1^, María Calbacho^1^, Pilar Martínez Sánchez^1^, José María Sánchez Pina^1^, Alejandro Martín García-Sancho^6^, Gloria Figaredo^1^, Laura Rufián^1,2^, Margarita Rodríguez^1,2^, Laura Carneros^1^, Carolina Martínez-Laperche^7^, Mariana Bastos-Oreiro^7^, Chongwu Wang^8^, María-Teresa Cedena^1^, Inmaculada Rapado^1^, Paula de Toledo^3^, Miguel Gallardo^1^, Antonio Valeri^1^, Rosa Ayala^1^, Joaquín Martínez-López^1^ and Santiago Barrio^1,2^.

The updated author contribution section appears below:

AJ-U, AM-M, MP, and SB designed the research. LR, AG-O, ER, PS and MR performed the experiments. AM-M, YR-H, SD, CW, and SB defined the bioinformatic pipeline and performed sequencing data analysis. AJ-U, MP, ER, TB, AR, MC, GF, M-TC, RA and JM-L provided patient samples and clinical data. Author MG contributed to the conception, design and manuscript revisions. All authors analyzed and interpreted the data. AJ-U, AM-M, MP, and SB wrote the manuscript which was approved by all authors.

The authors apologize for this error and state that this does not change the scientific conclusions of the article in any way. The original article has been updated.

