# Supplementary material for: Personalized monitoring of circulating tumor DNA with a specific signature of trackable mutations after chimeric antigen receptor T-cell therapy in follicular lymphoma patients
Source: Front Immunol. 2023 Jun 5;14:1188818. doi: 10.3389/fimmu.2023.1188818 (PMC10277746; doi:10.3389/fimmu.2023.1188818)

**Supplementary Figure 1**. Disease dynamics of each patient monitored by LiqBio-MRD. For each patient, two figures are shown: full image, with a left panel representing the baseline genotyping of lymph node (Solbio) and plasma (Liqbio) samples, a middle panel representing the VAF values of the different markers quantified by the ultrasensitive LiqBio-MRD test, and a right panel representing the LOD defined in healthy controls for every marker; MRD-value image, representing the mutation with the highest VAF at the sampling time-point.


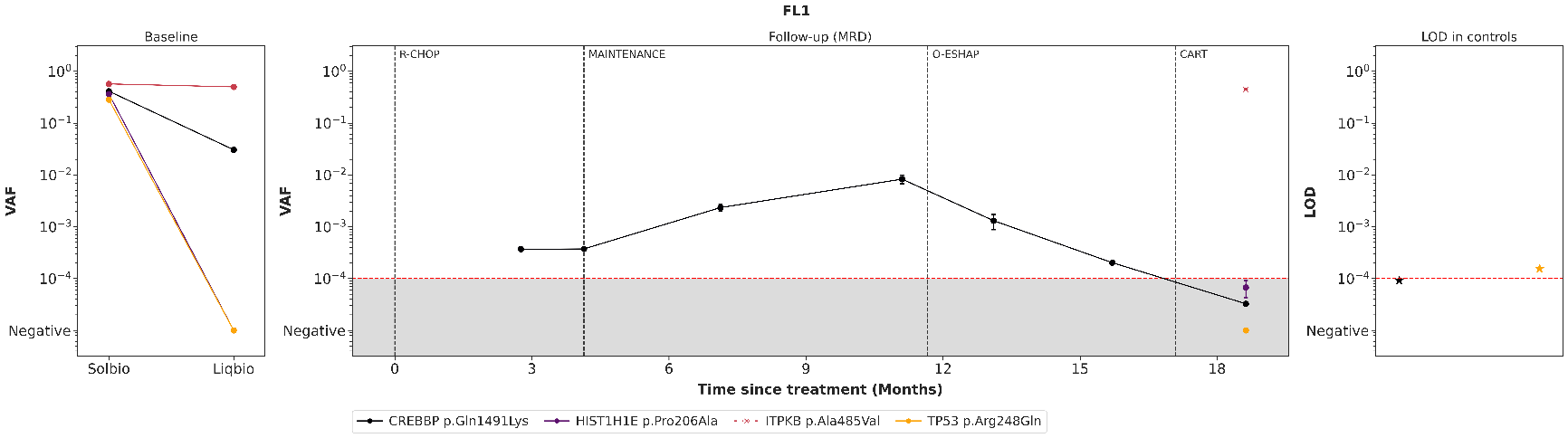


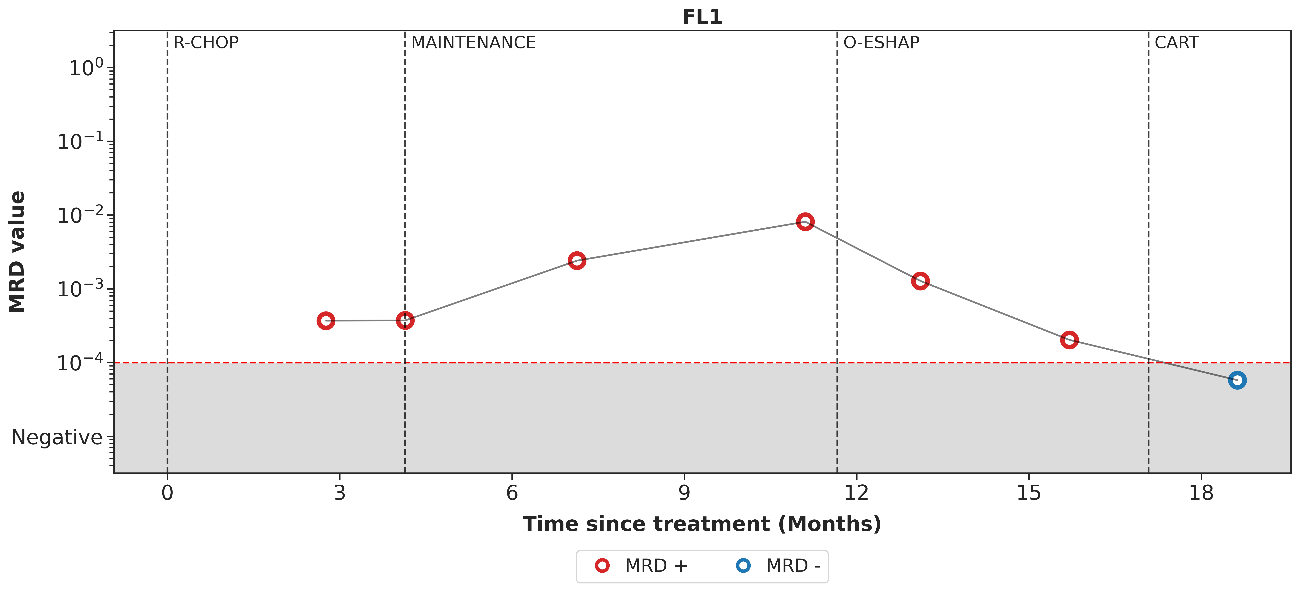


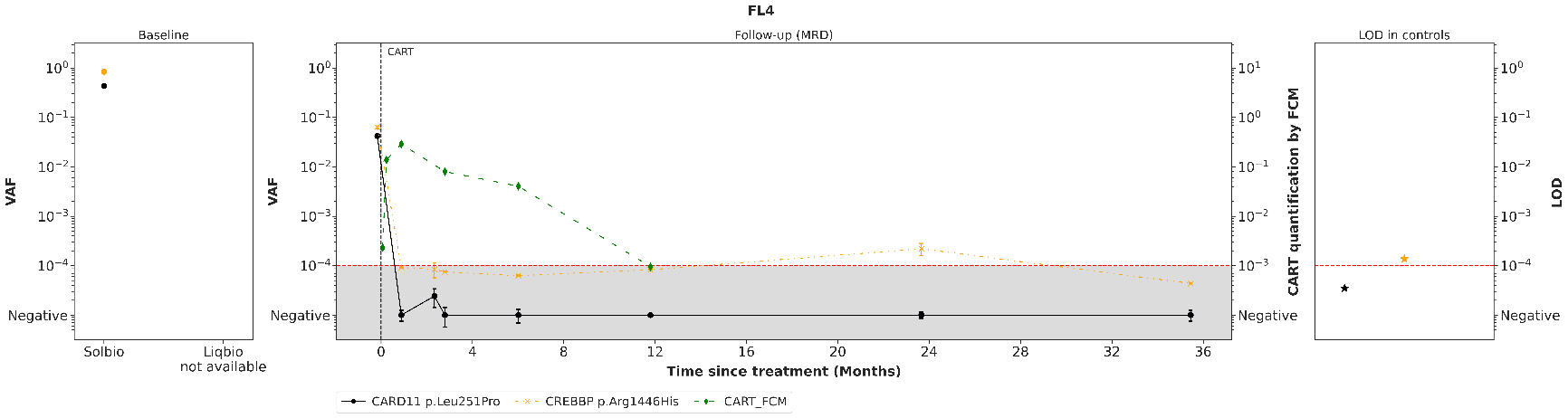


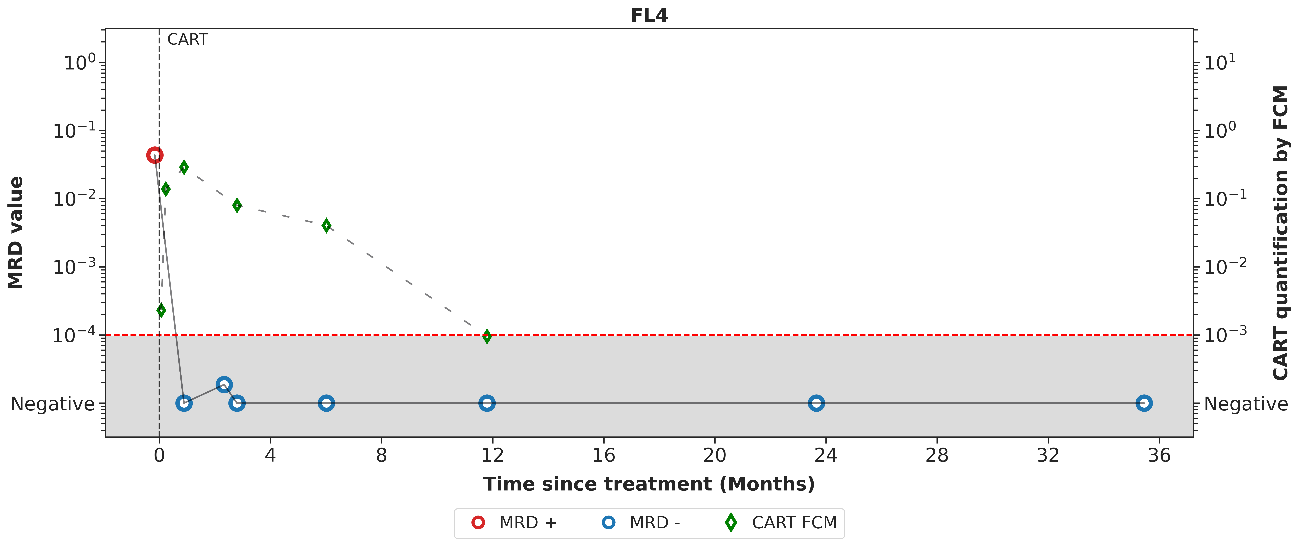


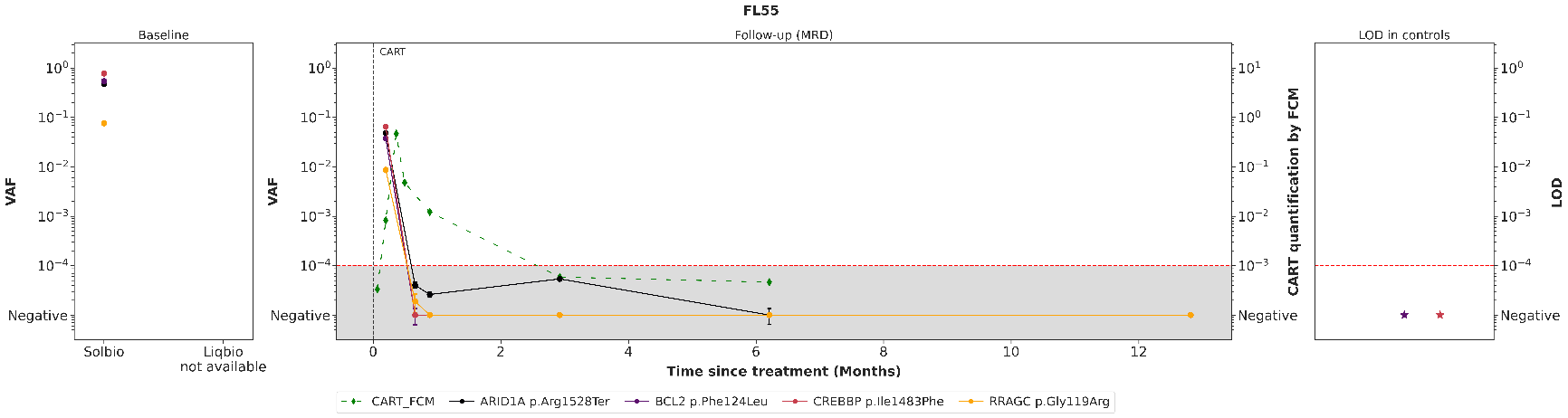


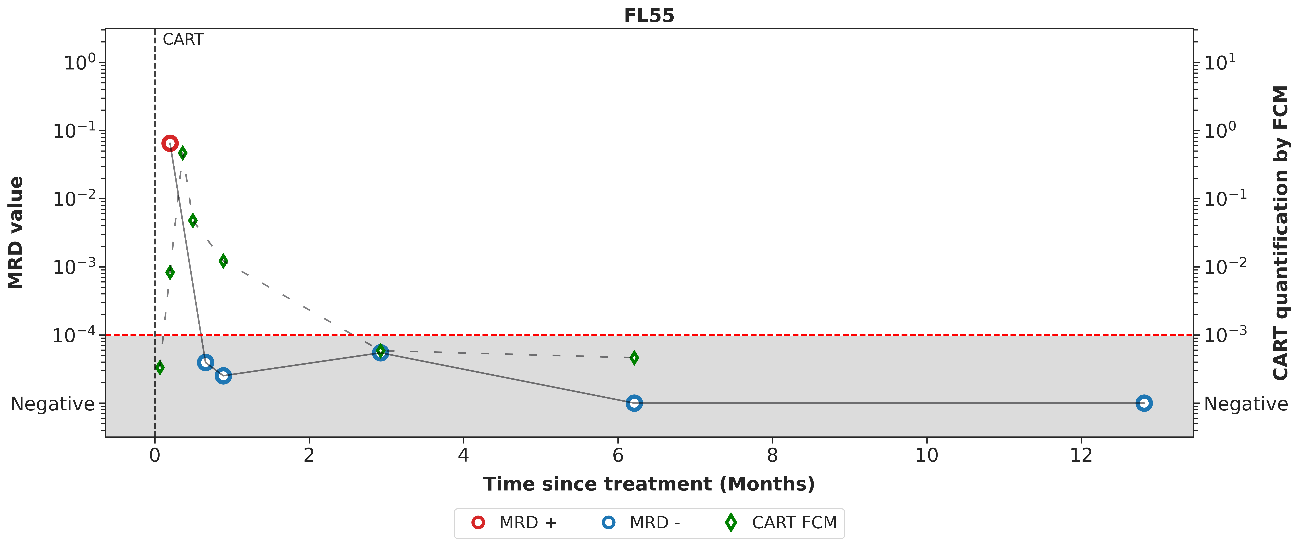


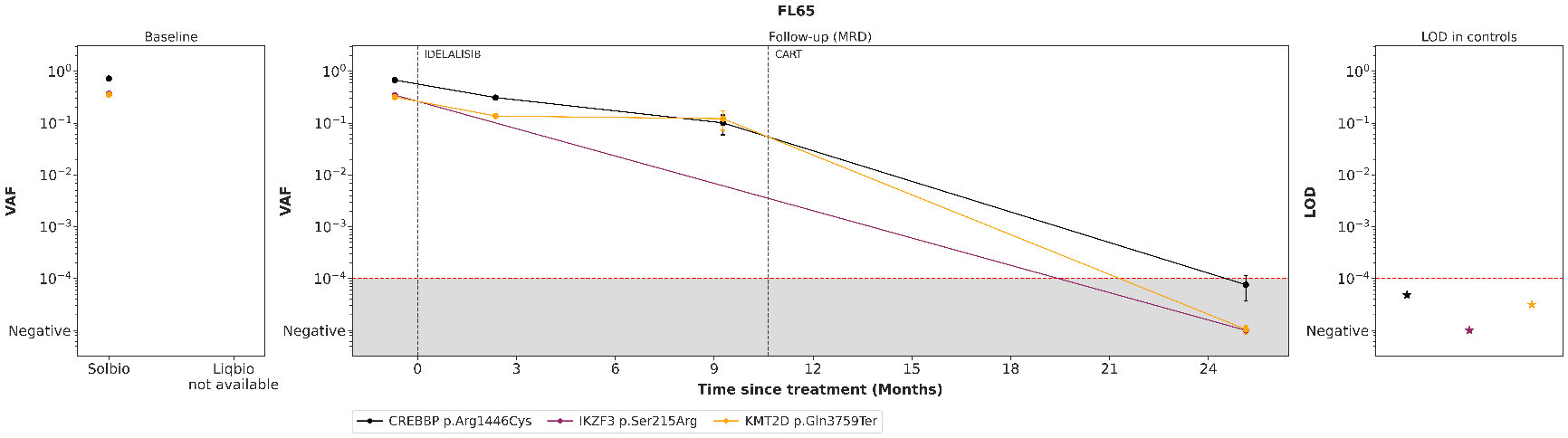


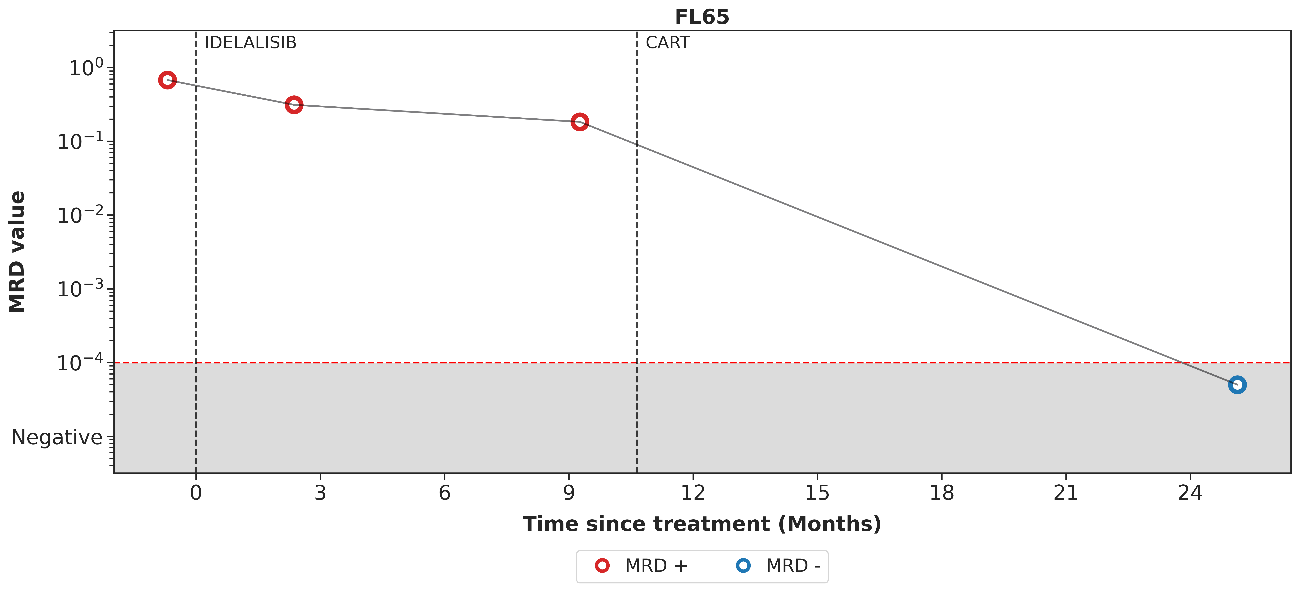


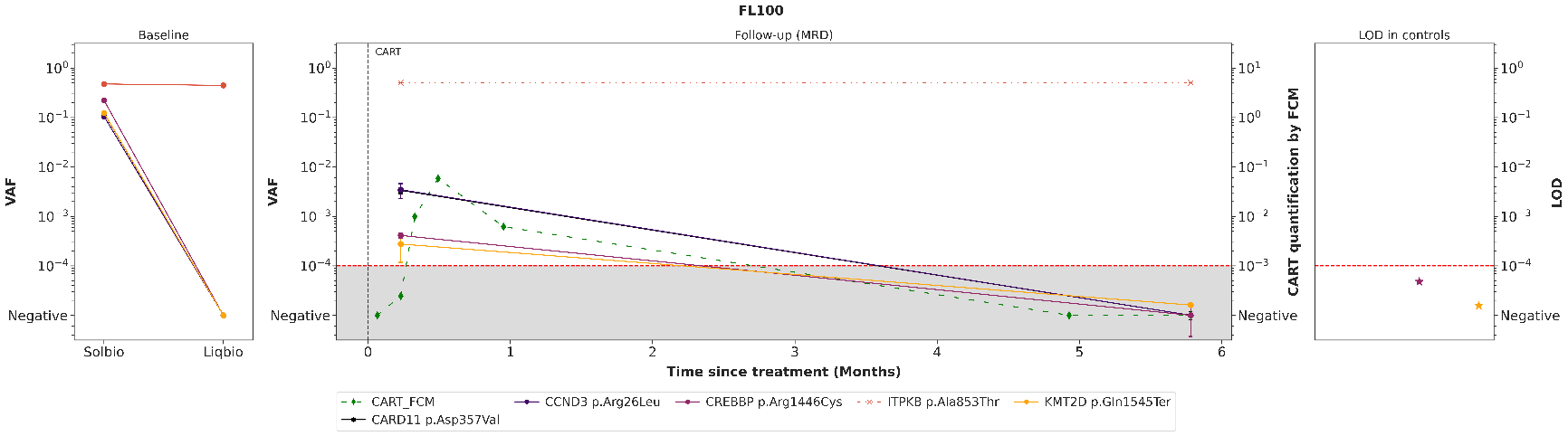


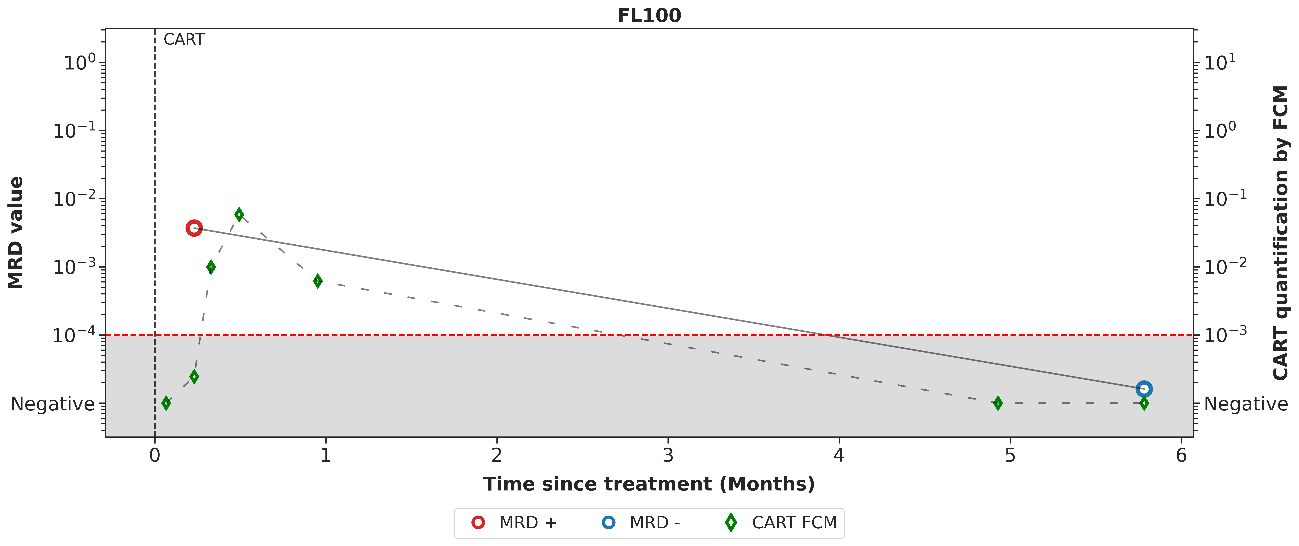


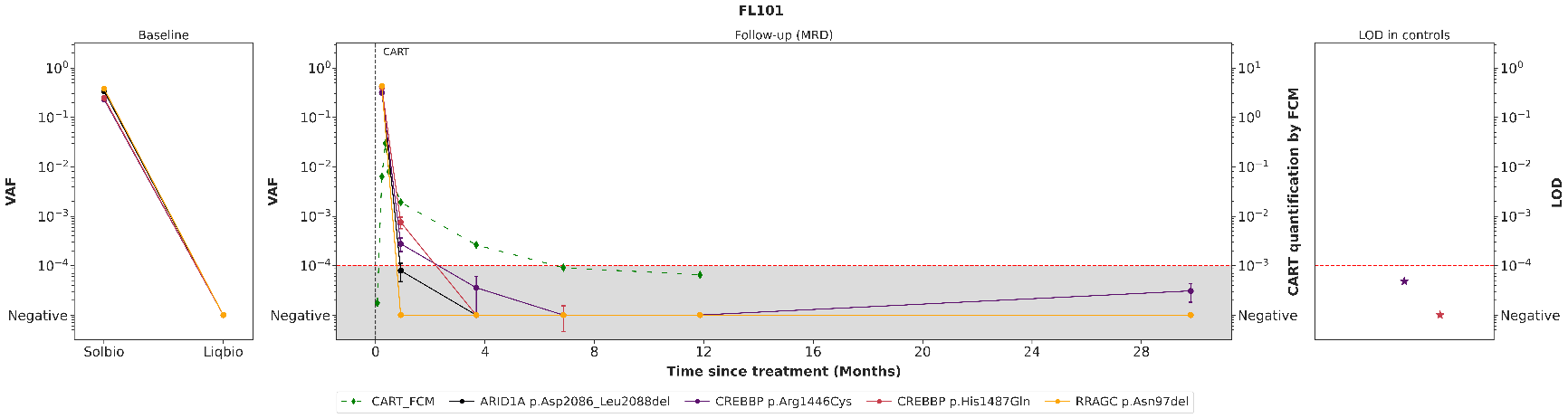


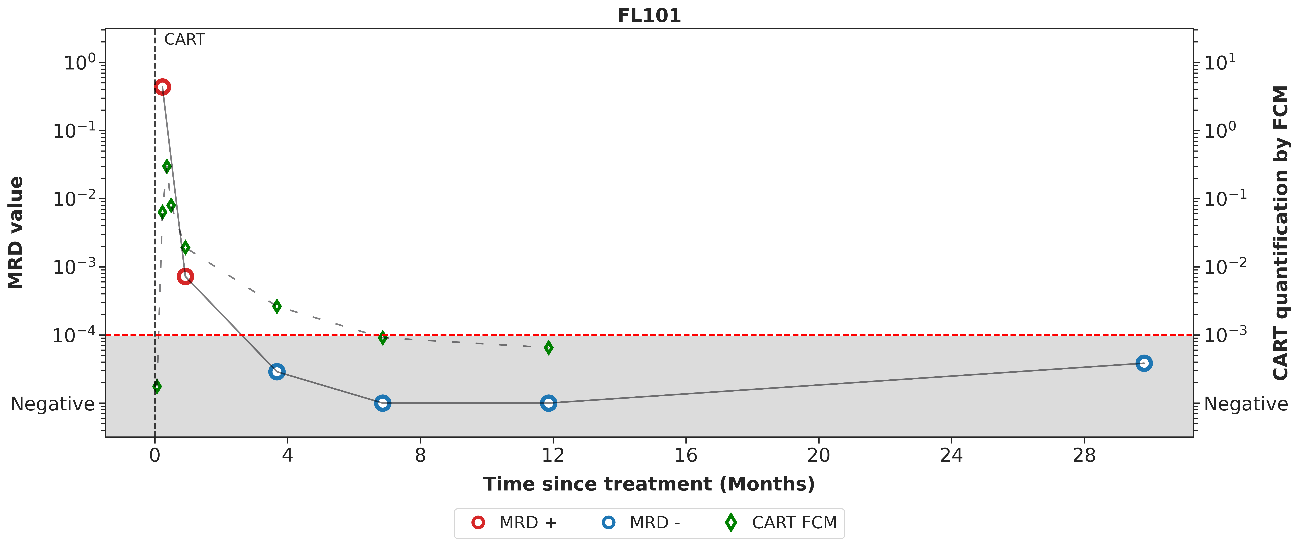


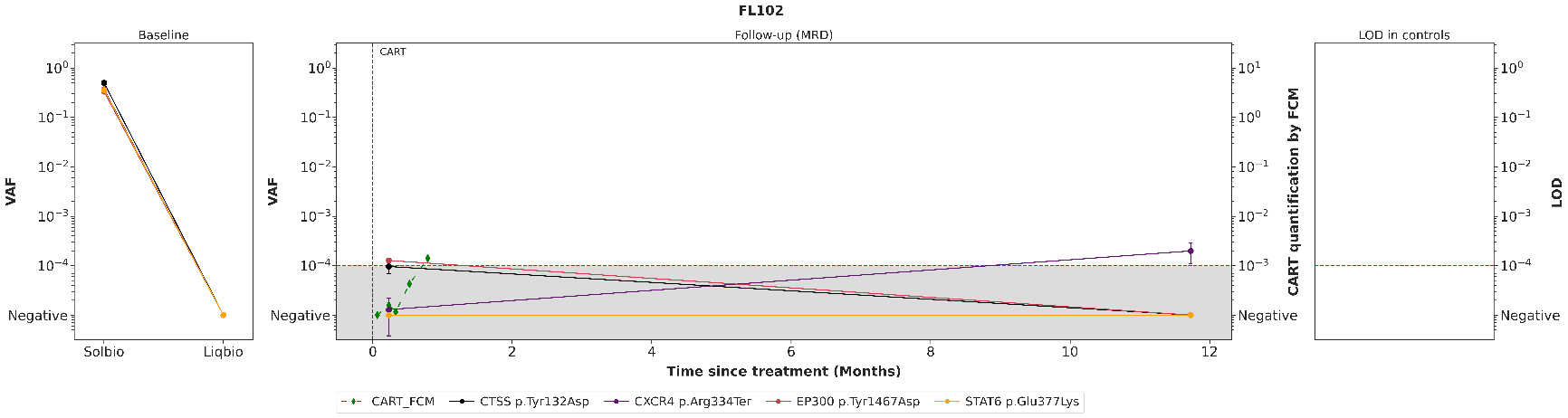


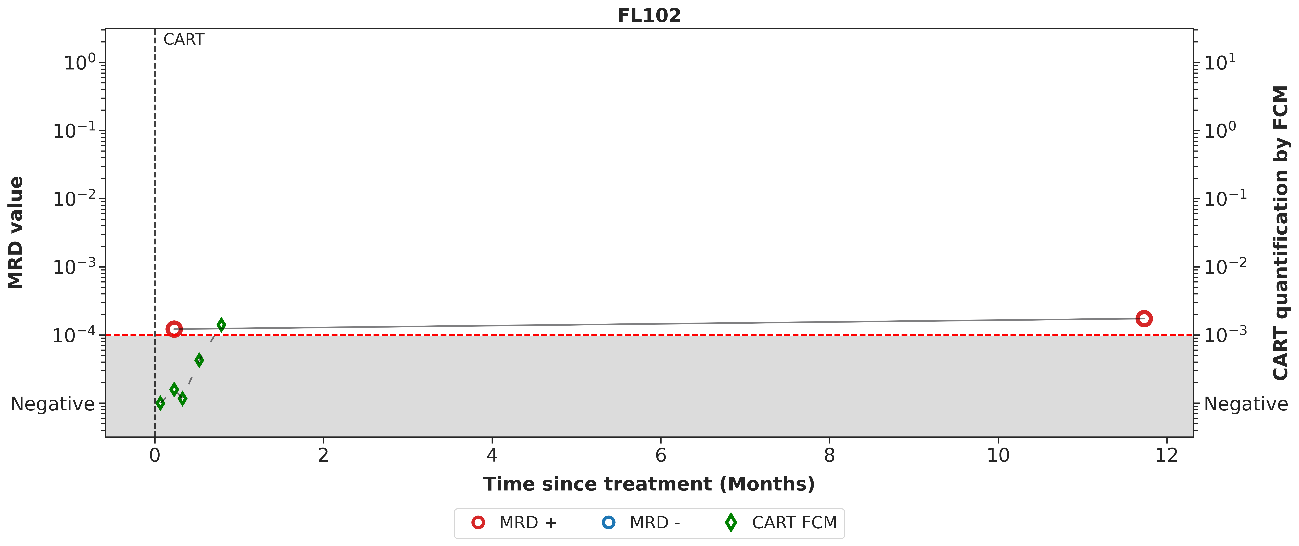


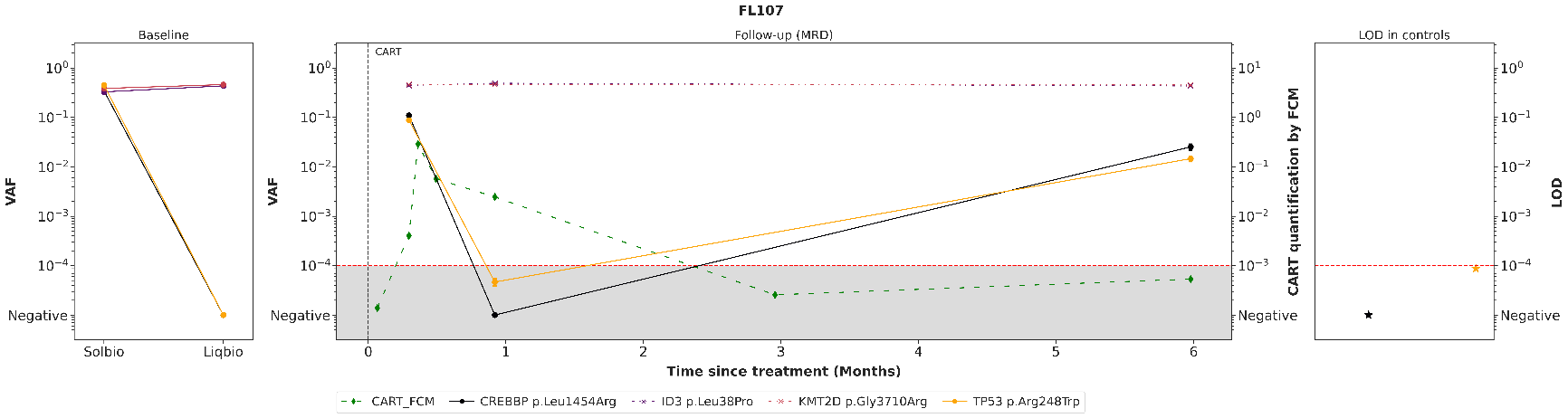


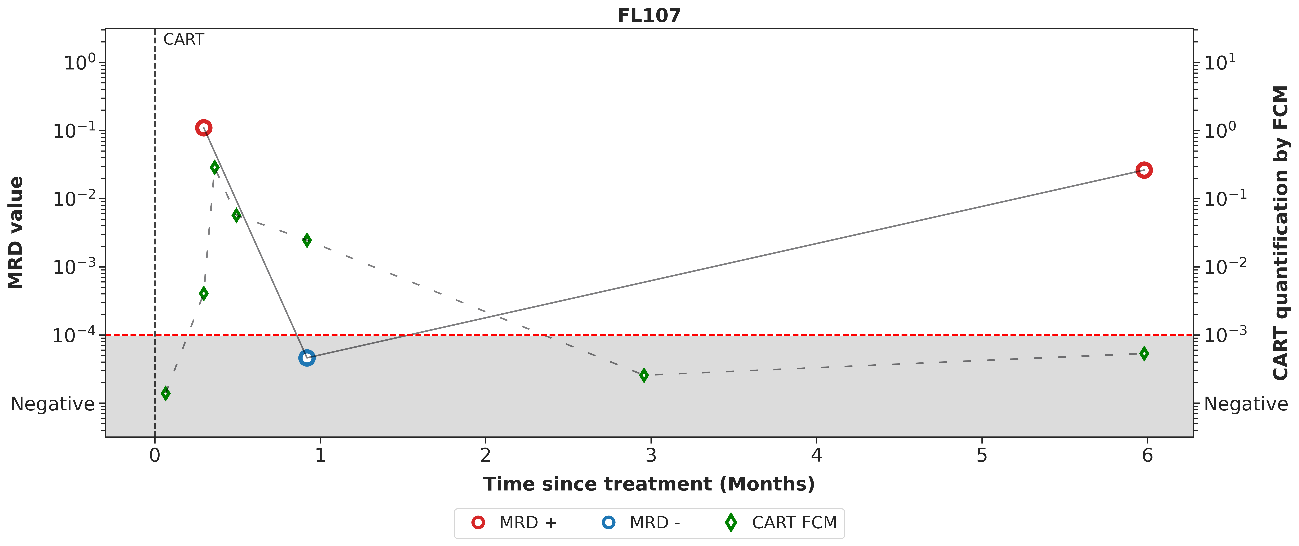


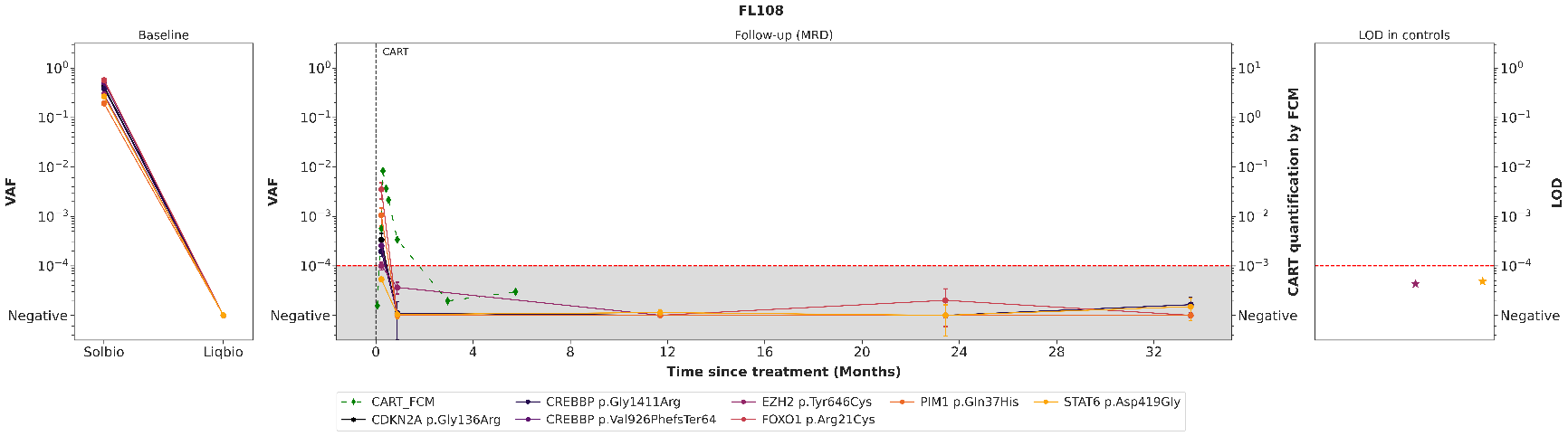


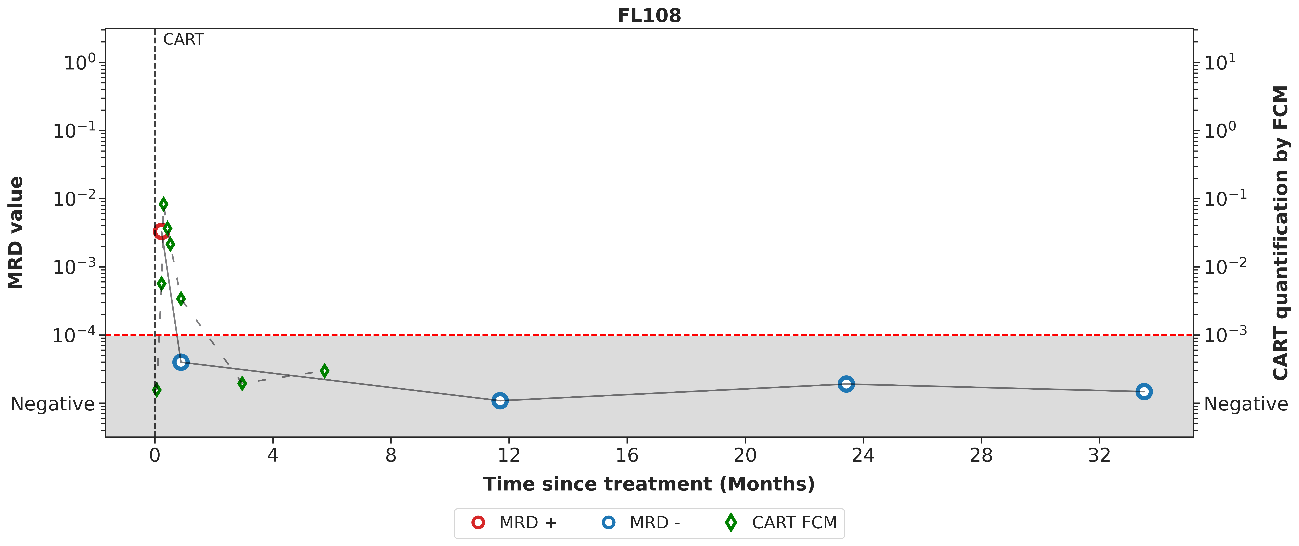


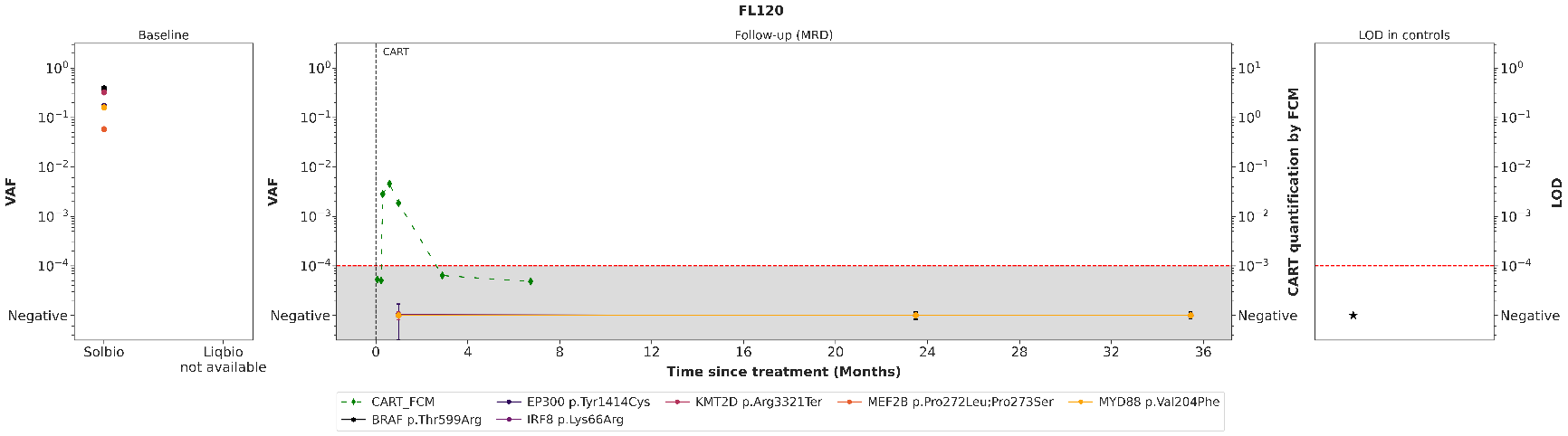


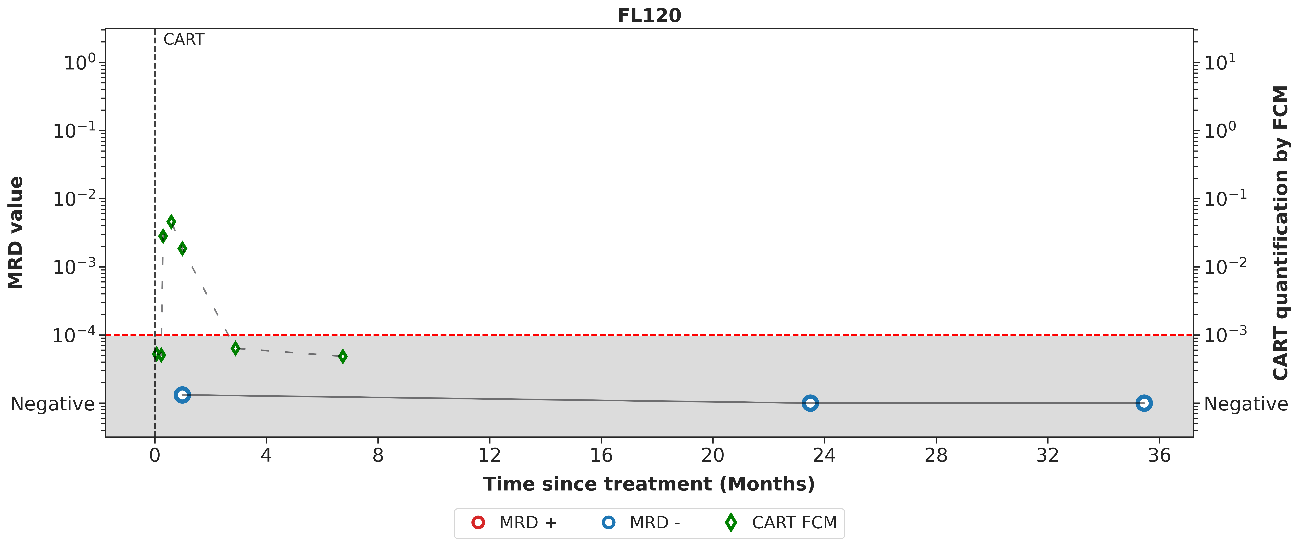


**Supplementary Figure 2**. Dynamics of CAR T-cells levels quantified by FCM (Y-axis) in the same patients, against the month since CAR T-cells infusion (X-axis), zooming in the first month. Patients that progressed are represented in red, CR and PR are represented in blue.


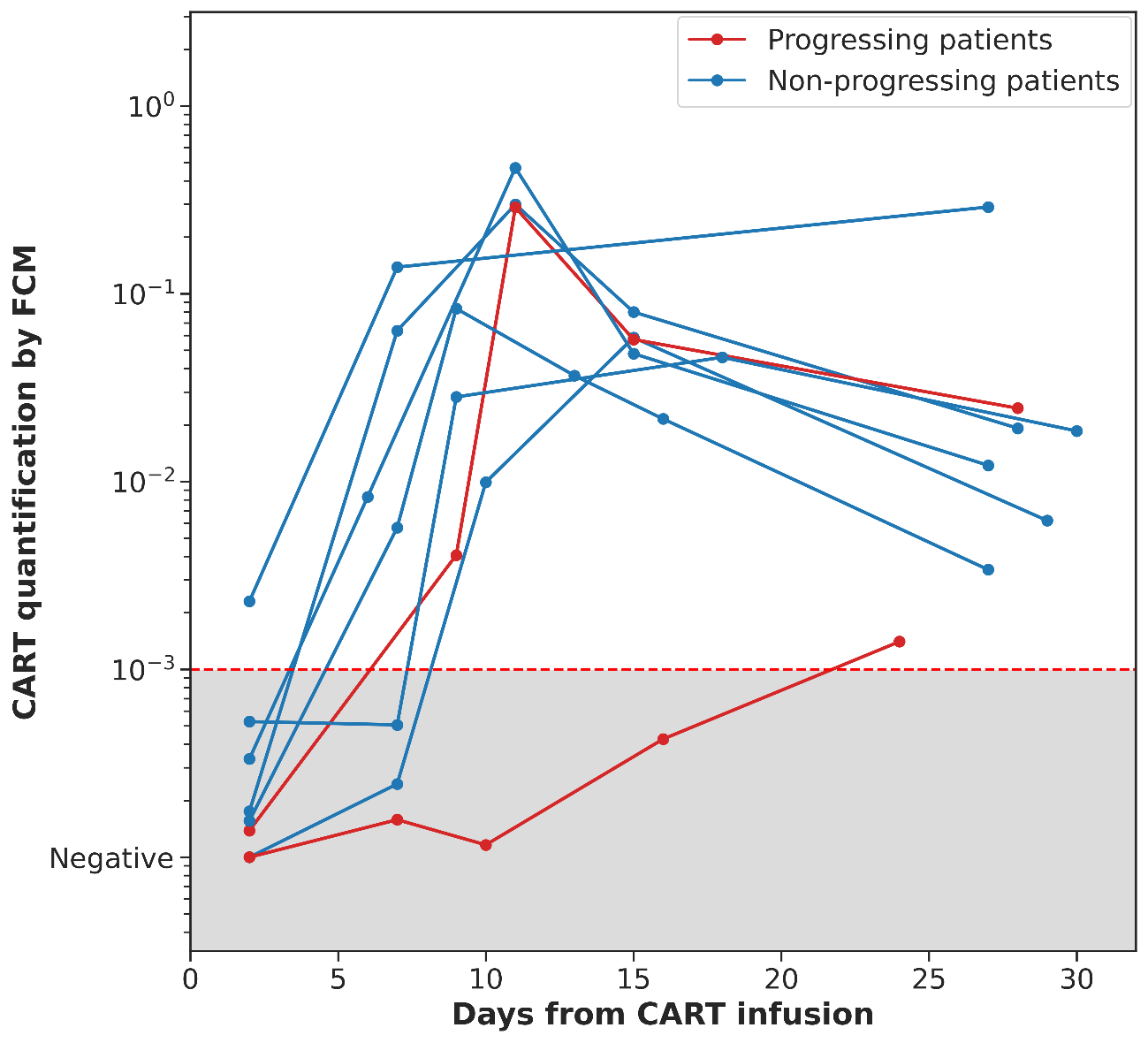


**Supplementary Figure 3**. Correlation plot among the CAR T-cells levels quantified by FCM and qPCR. Color corresponds to each patient, thus being shared among samples of the same patient. The linear relationship between two datasets is calculated with the Pearson correlation coefficient, also showed with the plot.


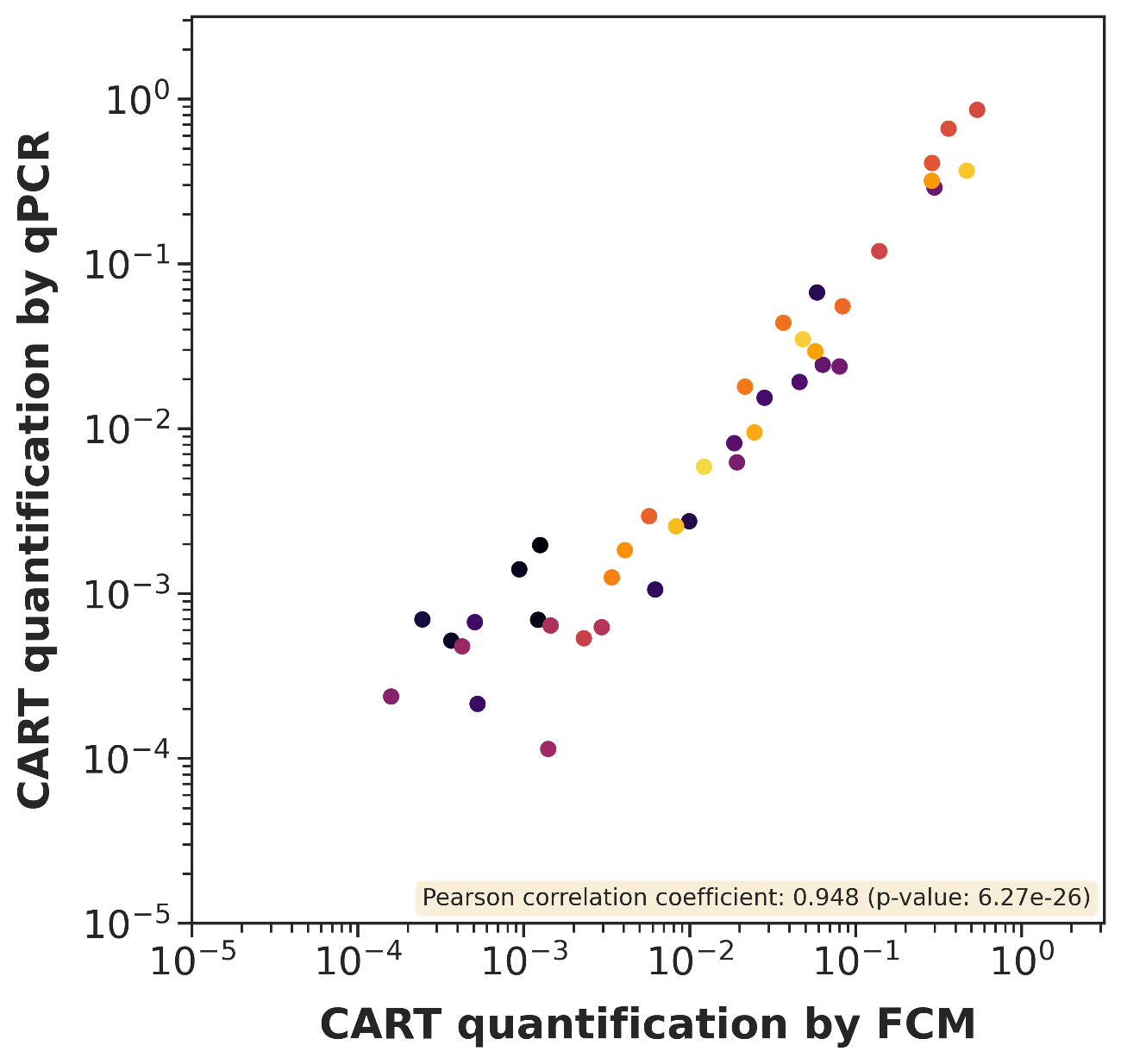

Supplement: Supplementary file 3 [file DataSheet_1.docx]
